# Novel Role of NOX in Supporting Aerobic Glycolysis in Cancer Cells with Mitochondrial Dysfunction and as a Potential Target for Cancer Therapy

**DOI:** 10.1371/journal.pbio.1001326

**Published:** 2012-05-08

**Authors:** Weiqin Lu, Yumin Hu, Gang Chen, Zhao Chen, Hui Zhang, Feng Wang, Li Feng, Helene Pelicano, Hua Wang, Michael J. Keating, Jinsong Liu, Wallace McKeehan, Huamin Wang, Yongde Luo, Peng Huang

**Affiliations:** 1 Department of Molecular Pathology, The University of Texas MD Anderson Cancer Center, Houston, Texas, United States of America; 2 State Key Laboratory of Oncology in Southern China, Sun Yat-Sen University Cancer Center, Guangzhou, China; 3 Department of GI Medical Oncology, The University of Texas MD Anderson Cancer Center, Houston, Texas, United States of America; 4 Department of Leukemia, The University of Texas MD Anderson Cancer Center, Houston, Texas, United States of America; 5 Department of Pathology, The University of Texas MD Anderson Cancer Center, Houston, Texas, United States of America; 6 Proteomics and Nanotechnology Laboratory, Center for Cancer and Stem Cell Biology, Institute of Biosciences and Technology, Texas A&M University Health Science Center, Houston, Texas, United States of America,; University of Wuerzburg, Germany

## Abstract

Elevated aerobic glycolysis in cancer cells (the Warburg effect) may be attributed to respiration injury or mitochondrial dysfunction, but the underlying mechanisms and therapeutic significance remain elusive. Here we report that induction of mitochondrial respiratory defect by tetracycline-controlled expression of a dominant negative form of DNA polymerase γ causes a metabolic shift from oxidative phosphorylation to glycolysis and increases ROS generation. We show that upregulation of NOX is critical to support the elevated glycolysis by providing additional NAD+. The upregulation of NOX is also consistently observed in cancer cells with compromised mitochondria due to the activation of oncogenic Ras or loss of p53, and in primary pancreatic cancer tissues. Suppression of NOX by chemical inhibition or genetic knockdown of gene expression selectively impacts cancer cells with mitochondrial dysfunction, leading to a decrease in cellular glycolysis, a loss of cell viability, and inhibition of cancer growth in vivo. Our study reveals a previously unrecognized function of NOX in cancer metabolism and suggests that NOX is a potential novel target for cancer treatment.

## Abstract 2

NAD(P)H oxidase plays a role in cancer metabolism by providing NAD^+^ to support increased glycolysis.

## Author Summary

Glycolysis is a cytoplasmic metabolic process that produces energy from glucose. In normal cells, the rate of glycolysis is low, and glycolysis products are further processed in the mitochondria via oxidative phosphorylation, a very efficient energy-producing process. Cancer cells, however, display higher levels of glycolysis followed by cytoplasmic fermentation, and reduced levels of oxidative phosphorylation. It was thought that increased glycolysis is associated with mitochondrial dysfunction, but how these phenomena are functionally linked was not known. Understanding how these processes are regulated will be essential for developing more effective anti-cancer therapies. Here, we show that induction of mitochondrial dysfunction by either genetic or chemical approaches results in a switch from oxidative phosphorylation to glycolysis. We further show that NADPH oxidase (NOX), an enzyme known to catalyze the oxidation of NAD(P)H, also plays a critical role in supporting increased glycolysis in cancer cells by generating NAD^+^, a substrate for one of the key glycolytic reactions. Inhibition of NOX leads to inhibition of cancer cell proliferation in vitro and suppression of tumor growth in vivo. This study reveals a novel function for NOX in cancer metabolism, explains the increased glycolysis observed in cancer cells, and identifies NOX as a potential anti-cancer therapeutic target.

## Introduction

Development of selective anticancer agents based on the biological differences between normal and cancer cells is essential to improve therapeutic selectivity. Increased aerobic glycolysis and elevated oxidative stress are two prominent biochemical features frequently observed in cancer cells. A metabolic shift from oxidative phosphorylation in the mitochondria to glycolysis in the cytosol in cancer was first described some 80 years ago by Otto Warburg, who later considered such metabolic changes as “the origin of cancer” resulting from mitochondrial respiration injury [1]. It is now recognized that elevated glycolysis is a characteristic metabolism in many cancer cells. In fact, active glucose uptake by cancer cells constitutes the basis for fluorodeoxyglucose-positron emission tomography (FDG-PET), an imaging technology commonly used in cancer diagnosis. In addition, cancer cells exhibit elevated generation of reactive oxygen species (ROS), which disturb redox balance leading to oxidative stress [2]. However, despite these long-standing observations and clinical relevance, the biochemical/molecular mechanisms responsible for such metabolic alterations and their relationship with mitochondrial respiratory dysfunction remain elusive. Identification of the major molecular players involved in the metabolic switch in the context of mitochondrial dysfunction in cancer cells is important for understanding the underlying mechanisms and developing more effective treatment strategies.

For many years, studies of mitochondrial respiratory defect usually involve the use of ρ° cells, in which mitochondrial DNA (mtDNA) deletion is generated by chronic exposure of cells to the DNA-intercalating agent ethidium bromide [3]. While successful, the use of ρ° cells generated by this method as a model for metabolic study has potential complications due to possible nuclear DNA damage by ethidium bromide and thus may compromise data interpretation [4]. To investigate the relationship between mitochondrial dysfunction and alterations of cellular metabolism, it is important to establish a model system in which the mitochondrial function can be regulated without significant impact on the nuclear genome. Mitochondrion DNA polymerase gamma (POLG) is a key enzyme responsible for the replication of mtDNA [5],[6], which encodes for 13 critical components of the respiratory chain. Thus, it is possible to specifically impact the mitochondrial respiratory function by selectively suppressing POLG, which is not involved in nuclear DNA replication. Indeed, a dominant negative form of POLG (POLGdn), which contains a point mutation (D1135A) in the coding sequence, has been previously identified and demonstrated to have a negative impact on mtDNA replication, causing respiratory defect after transfection [7],[8]. Thus, it is possible to use a gene transfection strategy to selectively impact mitochondrial function without affecting nuclear DNA.

NOX are a group of membrane-associated enzymes capable of oxidizing NADPH or NADH to NADP^+^ or NAD^+^, leading to generation of superoxide by one-electron reduction of oxygen [9]. There are seven members of the NOX family in the human genome with unique patterns of cellular expression and conserved structure. They include NOX1 (also called Mox1), NOX2 (gp91phox), NOX3, NOX4, NOX5, and the dual oxidases DUOX1 and DUOX2. Activation of most NOX complexes requires proper assembling of multiple protein components, including p22phox (CYBA), Rac-GTPase (Rac1 and Rac2), p47phox, p67phox, p40phox, NOX organizer 1 (NOXO1), and NOX activator 1 (NOXA1) [10],[11]. NOX enzymes have been implicated in host defense, regulation of gene expression, ROS generation, and redox signaling [12]. In many cancers, NOX activities are increased and mRNAs are overexpressed [13]–[16], but the precise functions of NOX in cancer cell metabolism remain unclear.

In the current study, we adapted the molecular strategy using POLGdn to generate a tetracycline-inducible cell system as previously described [8] and used this model system and cancer cells with compromised mitochondrial respiration to investigate the relationship between mitochondrial respiratory dysfunction and metabolic alterations and to identify the key molecular players in the metabolic switch from oxidative phosphorylation to aerobic glycolysis. We discovered an unexpected metabolic function of NOX, which is important for maintaining high glycolytic activity in cells with mitochondrial respiratory dysfunction. We also found that cancer cells with mitochondrial dysfunction due to the expression of oncogenic Ras or a loss of p53 consistently exhibited elevation in NOX activity and were highly sensitive to NOX suppression. Importantly, the significant increase in the expression of p22phox, a main component of NOX complex, was found in human pancreatic carcinoma, where K-Ras aberrant activation is prevalent. Furthermore, suppression of NOX exhibited significant antitumor activity in vivo, suggesting that NOX can be a potential target for cancer therapy.

## Results

### POLGdn Expression Leads to Mitochondrial Dysfunction and Elevated Glycolysis

**Figure 1:**
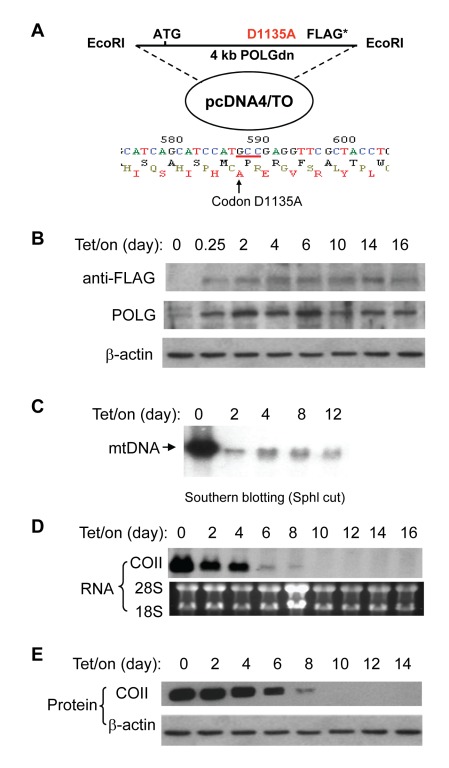
POLGdn expression led to the depletion of mtDNA-encoded respiratory chain components. (A) POLGdn-pcDNA4/TO construct and nucleotide sequencing analysis confirming the D1135A mutation. (B) Induction of POLGdn expression by doxycycline. T-Rex293 cells carrying POLGdn construction were incubated with doxycycline at an indicated time point. POLGdn expression was detected by anti-FLAG antibody, while both the endogenous POLG and POLGdn proteins were detected by anti-POLG antibody using Western blot assay. (C) Dramatic decrease of mtDNA by expression of POLGdn. Southern blot assay was used to measure mtDNA content. 10 µg total cellular DNA (including genomic DNA and mtDNA) from each sample was digested with SphI to linealize the circular mtDNA, followed by gel electrophoresis. ^32^P-labeled mitochondrial COII DNA fragment was used as a probe to detect mtDNA. (D) Assay of mtDNA-encoded COII RNA expression by northern blot analysis. (E) Detection of mitochondrial DNA-encoded COII protein by Western blot assay.

To investigate the relationship between mitochondrial dysfunction and metabolic changes, we first established an experimental cell model where the mitochondrial respiratory status can be regulated via a tetracycline-inducible system. This model is based on the fact that mtDNA replication is catalyzed by DNA polymerase γ (POLG) and that POLGdn can abolish mtDNA replication leading to respiration defects [7],[17]. A 4-kb DNA fragment containing the full-length POLGdn gene with a D1135A mutation was constructed into a mammalian expression vector pcDNA4/TO (Figure 1A), which was then transfected into human T-Rex 293 cells to generate a Tet/on inducible system as described in the Materials and Methods. As shown in Figure 1B, in the absence of doxycycline (Tet/off), the endogenous POLG was readily detected by POLG antibody, but the expression of exogenous POLGdn was not detectable (absence of the FLAG signal), suggesting that this experimental system was tightly controlled without detectable leakage. Addition of doxycycline (Tet/on) to the culture medium induced the expression of POLGdn (detected by anti-FLAG), which remained expressed for over 2 wk in the presence of doxycycline. The induced expression of POLGdn was also evident by the increase of the band intensity detected by anti-POLG antibody (Figure 1B).

The expression of POLGdn led to a severe decrease of mtDNA synthesis, as evidenced by a dramatic decrease of mtDNA 2 d after doxycycline induction (Figure 1C). To determine the mtDNA-encoded gene expression, Northern blot analysis was used to measure the level of mtDNA-encoded cytochrome c oxidase subunit II (COII) RNA. Figure 1D showed that the expression of COII RNA was decreased on day 2, almost depleted on day 6, and disappeared by day 10 after POLGdn induction. Western blot analysis showed a corresponding depletion of COII protein in a time-dependent manner (Figure 1E).

**Figure 2:**
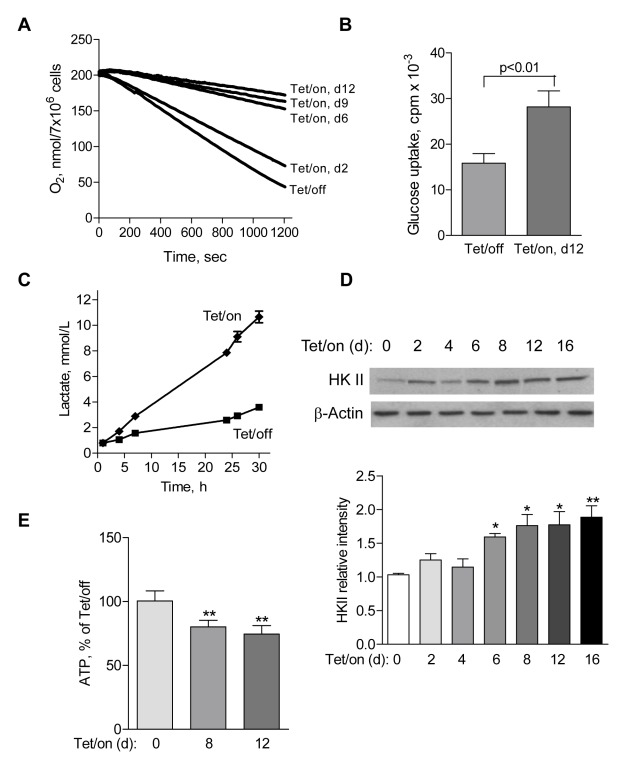
Suppression of mitochondrial respiration by POLGdn expression led to an elevation of glycolysis. (A) Time-dependent decrease in cellular oxygen consumption following POLGdn expression. Reduction of oxygen consumption was observed as early as 2 d after POLGdn expression, and the cells dramatically decreased their ability to consume oxygen with prolonged POLGdn expression. (B) Increased glucose uptake in POLGdn-expressing cells (Tet/on, d12). Cells (2×10^6^) were incubated in 5 ml glucose-free RPMI1640 medium for 2 h, followed by incubation with 0.2 µCi/mL ^3^H-2-deoxyglucose for 1 h. Cellular uptake of ^3^H-2-deoxyglucose was determined by liquid scintillation counting after the cells were washed two times with PBS. Error bars, ±SD. *p*<0.01 (*n* = 3). (C) Increased lactate generation in Tet/on cells. Lactate in Tet/off and Tet/on (day 12) cells was measured at the indicated time points after changing to fresh culture medium. (D) Increased protein level of hexokinase II (HKII) following POLGdn expression. Upper panels show representative HKII protein by Western blotting assay at the indicated days after POLGdn induction by doxycycline. Lower panels show quantification of Western blot results using scanning and ImageJ software. Results are expressed as integrated optical density. Each sample was normalized to β-actin content. Each bar represents the mean ± SEM of three independent experiments. * *p*<0.05; ** *p*<0.01. (E) Comparison of cellular ATP levels in cells with or without POLGdn expression. Cellular ATP contents in Tet/on cells (days 8 and 12) were measured and compared with the Tet/off cells. Error bars, ±SD (** *p*<0.01 and *n* = 3).

To evaluate the metabolic alterations subsequent to POLGdn induction, we first measured cellular oxygen consumption as an indicator of mitochondrial respiratory capacity [18]. Expression of POLGdn caused a time-dependent decrease in oxygen consumption, detectable on day 2 and dramatically decreased on day 6 (Figure 2A). Associated with this decrease in mitochondrial respiration, the Tet/on cells became highly glycolytic, as evidenced by a significant increase in glucose uptake (Figure 2B) and elevated production of lactate, a metabolic product of glycolysis (Figure 2C). Consistent with the active glycolysis, the Tet/on cells showed an upregulation of hexokinase II (HKII), a rate-limiting enzyme of the glycolytic pathway (Figure 2D). This increase in glycolysis seemed effective in compensating the decrease of ATP production in the mitochondria, since the overall cellular ATP levels only decreased moderately (Figure 2E), even on day 12 when respiration was severely suppressed.

**Figure 3:**
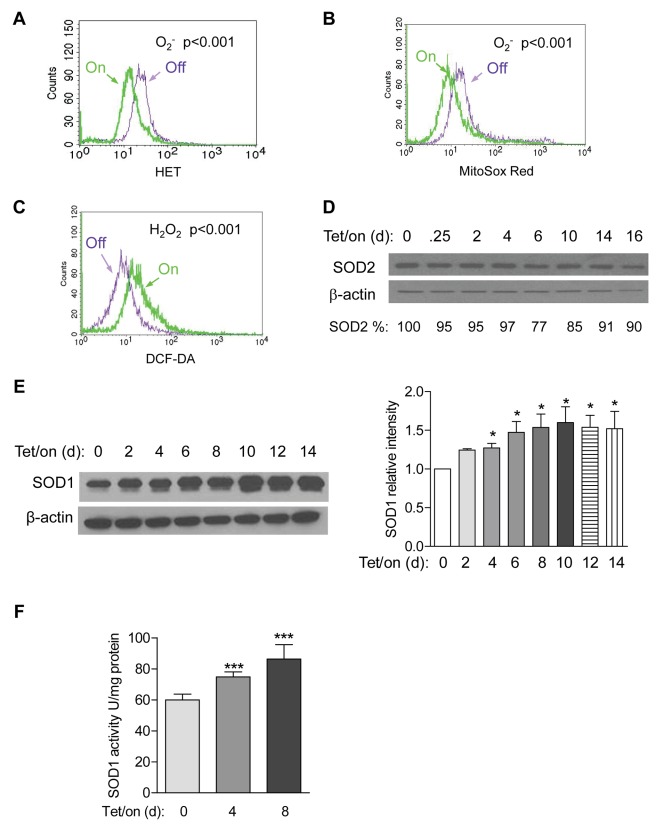
Alterations in ROS generation and SOD expression in cells with mitochondrial defect induced by POLGdn. (A) Lower cellular O_2_^−^ in POLGdn-expressing cells (Tet/on, day 12). O_2_^−^ was detected by flow cytometry using 200 ng/ml HET as fluorescent dye (*p*<0.001, Tet/off versus Tet/on and *n* = 3). (B) Comparison of mitochondrial O_2_^−^ in cells with POLGdn expression (Tet/on day 12) or without POLGdn (Tet/off). Mitochondrial O_2_^−^ was detected by flow cytometry using 5 µm MitoSox Red as fluorescent dye (*p*<0.001, Tet/off versus Tet/on and *n* = 3). (C) Increase in cellular H_2_O_2_ level in POLGdn expressing Tet/on (day 12) cells. Cellular H_2_O_2_ was measured by flow cytometry using 4 µm DCF-DA as a fluorescent dye (*p*<0.001, Tet/off versus Tet/on, and *n* = 3). (D) Protein level of mitochondrial superoxide dismutase (SOD2) in cells at the indicated time points after POLGdn induction. SOD2 was assayed by Western blot analysis. β-actin was used as a loading control. (E) Protein level of cytosolic superoxide dismutase (SOD1) in cells at the indicated time points after POLGdn induction. Left panels show representative Western blots, and right panels show quantification of normalized SOD1 levels to β-actin controls from three independent experiments. Data are shown in mean ± SEM. * *p*<0.05. (F) Changes in SOD1 activity in cells at the indicated time points after POLGdn induction. Error bars, ±SD. *** *p*<0.001 (*n* = 3).

Since the mitochondrial respiratory chain is a major site of cellular ROS generation, we examined if induction of mitochondrial dysfunction by POLGdn could lead to a change in cellular ROS. Flow cytometry analysis of cells stained with hydroethidine (HET), a relatively specific chemical probe for superoxide (O_2_^−^) [19] showed that the cellular O_2_^−^ level was significantly decreased in the Tet/on cells at day 12 (Figure 3A). Consistently, MitoSox Red, a mitochondrial O_2_^−^ indicator, revealed a significant decrease in mitochondrial O_2_^−^ in the Tet/on cells (Figure 3B). In contrast, a general redox-sensitive probe DCF-DA, which detects cellular H_2_O_2_ and other ROS, revealed a significant increase in cellular ROS in the Tet/on cells (Figure 3C).

Because O_2_^−^ can be converted to H_2_O_2_ by the mitochondrial superoxide dismutases (SOD2) or the cytosolic SOD1, we tested the possibility that the increase in cellular H_2_O_2_ and the decrease in O_2_^−^ observed in the Tet/on cells might be a consequence of altered SOD activity. Western blot analysis showed that the mitochondrial SOD2 remained unchanged (Figure 3D), whereas the basal expression of cytosolic SOD1 was abundant in the Tet/off cells and was further increased by day 4 after POLGdn induction (Figure 3E). Concurrently, SOD1 activity was also increased (Figure 3F). Taken together, these data suggest that the increase in cellular ROS detected by DCF-DA was most likely due to elevated generation of O_2_^−^ outside the mitochondria, and such cytosolic O_2_^−^ was then converted to H_2_O_2_ by the elevated SOD1.

### Upregulation of NOX Is Important to Maintain High Glycolytic Activity in Cells with Mitochondrial Dysfunction

**Figure 4:**
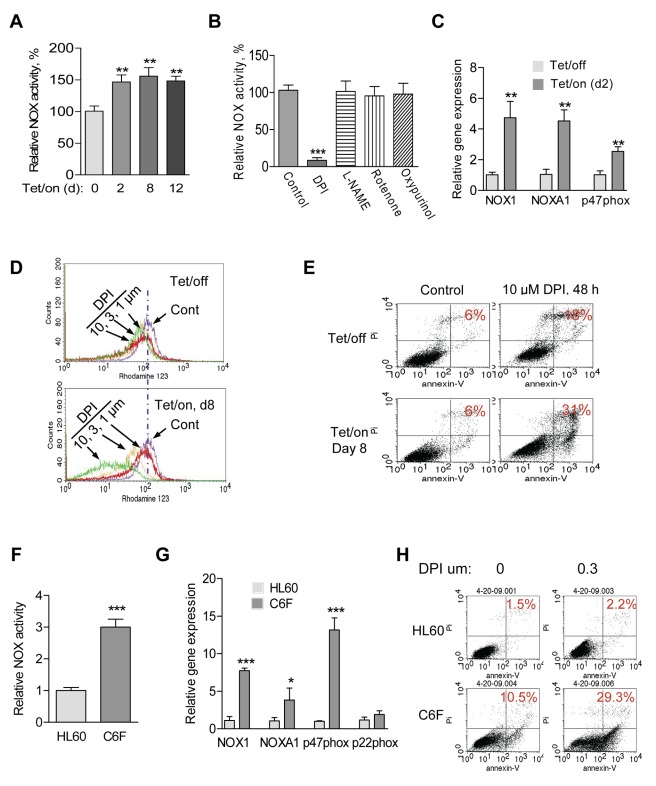
Cells with mitochondrial respiratory defects exhibit elevated NOX activity and are sensitive to NOX inhibition. (A) Increase of membrane-associated NOX activity in cells with mitochondrial respiratory defects after induction of POLGdn expression (Tet/on, 2, 8, 12 d). Error bars, ±SD. ** *p*<0.01 (*n* = 3). (B) Inhibition of NOX enzyme activity by DPI. The Tet/on cells (day 8) were treated with 10 µM DPI, 100 µM L-NAME, 20 µM rotenone, or 100 µM oxypurinol for 4 h, and the membrane-associated fractions were prepared for analysis of NOX activity. Error bars, ±SD. *** *p*<0.001 (*n* = 3). (C) Increase in mRNA expression of NOX family members in Tet/on (day 2) cells, measured by qRT-PCR analysis. Error bars, ±SD. ** *p*<0.01 (*n* = 3). (D) Comparison of changes in mitochondrial transmembrane potential in Tet/off and Tet/on cells treated with DPI. Cells were pre-induced by doxycycline for 7 d and then incubated with the indicated concentrations of DPI for 24 h. Mitochondrial transmembrane potential was measured by flow cytometry using Rhodamine-123 as a potential-sensitive dye. Cells without DPI treatment were marked as control (Cont). (E) Cells with mitochondrial respiratory defect (Tet/on, day 8) were more sensitive to DPI treatment (10 µM, 48 h) compared with the Tet/off cells. Cell viability was measured by annexin-V/PI assay. (F) Increase of NOX activity in mDNA-less HL60-C6F (C6F) cells. Mean ± SD. *** *p*<0.001 (*n* = 3). (G) Increase in NOX family mRNA expression in C6F cells, measured by qRT-PCR assay. Data are shown as mean ± SD of triplicate samples from two independent experiments. * *p*<0.5; *** *p*<0.001. (H) C6F cells were more sensitive to DPI treatment. HL60 and its derived C6F cells were treated with indicated concentration of DPI for 48 h. Cell viability was measured by annexin-V/PI assay.

Since NOX is a membrane-associated enzyme capable of generating ROS outside the mitochondria [20], we then measured the membrane-associated NOX activity of the Tet/on cells in comparison with Tet/off cells, using standard NOX assay described previously [21],[22]. The mitochondrial respiratory defective cells consistently exhibited a significant increase in NOX activity, which was detected 2 d after POLGdn induction and remained high as long as the cells were maintained in Tet/on stage (Figure 4A). This increase in NOX activity was inhibited by 10 µM diphenyleneiodonium (DPI), a known inhibitor of NOX [23], to less than 10% of the original NOX activity (Figure 4B). In contrast, pharmacologic inhibitors of other ROS-generating molecules including NOS inhibitor Nω-nitro-L-arginine mrthyl ester hydrochloride (L-NAME, 100 µM), the mitochondrial respiratory chain complex I inhibitor rotenone (20 µM), and the xanthine oxidase inhibitor oxypurinol (100 µM) showed no effect on the NOX activity assay (Figure 4B). To determine if the increased NOX activity was due to an increase in gene expression, we used semi-quantitative RT-PCR to evaluate possible changes in RNA expression of various NOX components and showed that the mRNA levels of NOX1, NOXA1, and p47^phox^ were significantly increased (Figure S1A–B, Text S1). Time-course analysis of NOXA1 and p47phox expression showed that the mRNA levels increased 2 d after Tet/on (Figure S1B), concurrent with the timing of NOX activity increase. This elevated gene expression was further quantitatively confirmed by qRT-PCR assay (Figure 4C).

The elevated NOX in cells with mitochondrial dysfunction induced by POLGdn suggests that NOX upregulation might be functionally important for these cells. We then used DPI to test if the POLGdn-expressing cells with mitochondrial respiratory defects might be more vulnerable to NOX inhibition. As shown in Figure 4D, the Tet/on cells were significantly more sensitive to DPI treatment than the Tet/off cells, evident by a substantial loss of mitochondrial integrity (ability to retain rhodamin-123), a decrease in cell viability (annexin-V/PI double staining, Figure 4E), and an inhibition of cell growth (Figure S1C). To test whether the elevated NOX gene expression and NOX activity were only limited to the POLGdn Tet/on system, we compared the respiration defective (ρ°) cell line HL60-C6F (C6F cells) [24] with its mitochondrial competent parental HL60 cell line and observed that the mitochondrial respiration-defective C6F cells showed a significant increase in NOX activity (Figure 4F) and elevated expression of NOX1, NOXA1, and p47phox (Figure 4G). Consistently, C6F cells were also more sensitive to DPI than the parental HL60 cells (Figure 4H).

**Figure 5:**
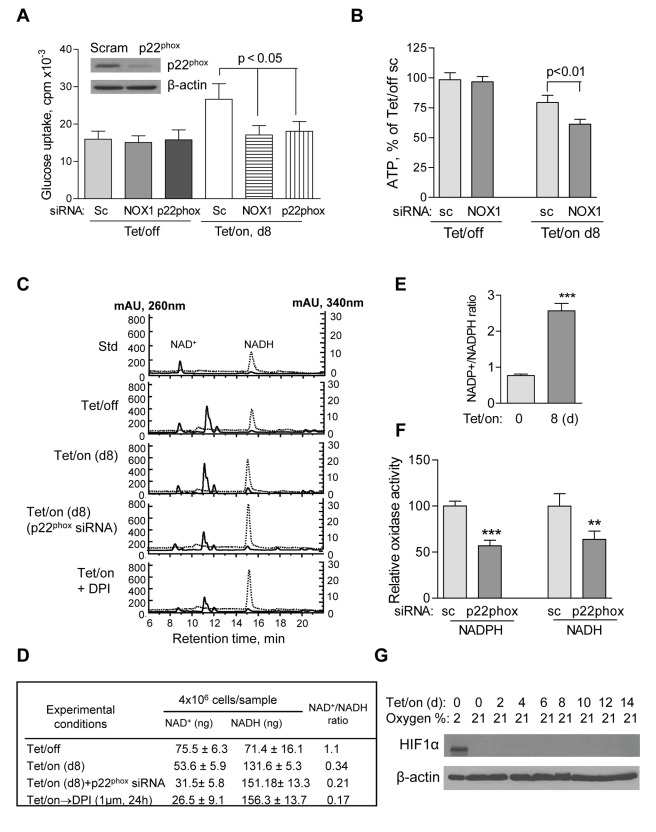
NOX supports glycolysis in cells with mitochondrial respiratory defects induced by POLGdn. (A) Inhibition of glucose uptake by siRNA knockdown of NOX1 or p22phox in cells with mitochondrial respiratory defects (Tet/on, day 8), but not in cells with intact mitochondria (Tet/off). *p*<0.05 (*n* = 3). Insert: Tet/off and Tet/on (at day 4) cells were transiently transfected with p22phox siRNA and the knockdown efficiency was detected by anti-p22phox antibody using Western blot. Non-targeting control siRNA (Scram or sc) was used as negative control. (B) Effect of NOX1 knockdown on ATP contents in cells with intact mitochondria (Tet/off) and cells with mitochondrial respiratory defect (Tet/on, day 8). *p*<0.01 (*n* = 4). (C) HPLC analysis of cellular NAD^+^ and NADH levels. Standard NAD^+^ and NADH were used as references, which were monitored simultaneously at 260 nm and 340 nm, respectively. A lower NAD^+^ content was detected in the Tet/on cells with mitochondrial respiratory dysfunction and higher glycolytic activity that consumed more of NAD^+^. Inhibition of NOX activity by p22^phox^ siRNA and DPI resulted in further decrease in cellular NAD^+^ level. (D) Quantitation of intracellular NAD^+^ and NADH in triplicate experiments, using HPLC method as described above. (E) Effect of POLGdn expression on cellular NADP+/NADPH ratio. *** *p*<0.001. (F) Knockdown of p22phox decreased NADPH/NADH oxidase activity. Comparing to the control siRNA knockdown in Tet/on (d8) cells, p22phox knockdown significantly decreased cellular NADPH/NADH oxidase activity using either NADHP or NADH as substrate. All error bars, ±SD. ** *p*<0.01; *** *p*<0.001 and *n* = 3. (G) HIF-1α is not stabilized following POLGdn induction. Protein level of HIF-1α in cells at the indicated time points after POLGdn induction was assayed by Western blot analysis. Tet/off cells with 2% oxygen were used as positive control. β-actin was used as a loading control.

The above observations suggest that the up-regulation of NOX might be important for the viability of cells with mitochondrial dysfunction. Since respiration-defective cells are highly dependent on glycolysis for cell survival, we tested the potential role of NOX on glucose metabolism by evaluating the effect of NOX knockdown on glucose uptake, ATP generation, and cellular NAD^+^ level. siRNA was used to specifically knockdown the expression of the critical NOX components NOX1 and p22phox in both Tet/on and Tet/off cells (Figure S2A and S2B). Interestingly, suppression of NOX by siRNA selectively impact cells with mitochondrial dysfunction (Tet/on), evidenced by a significant decrease in glucose uptake and reduced ATP generation, while cells with competent mitochondrial function (Tet/off cells) were not affected (Figure 5A–B), suggesting a potential role of NOX in glucose metabolism in cells that are highly glycolytic such as in the cells with mitochondrial defect. We further measured cellular NADH and NAD^+^ contents in the Tet/off and Tet/on cells in the presence and absence of NOX inhibition. As shown in Figure 5C, NADH and NAD^+^ were separated by HPLC and eluted at 9 min and 15 min, respectively. The chemical identities of NADH and NAD^+^ in these HPLC peaks were collected and confirmed by mass spectrometry analysis (Figure S2C and S2D). Quantitative analysis showed that the expression of POLGdn (Tet/on) caused a decrease in NAD^+^ by approximately 30% (75.5→53.6 ng/4×10^6^ cells; Figure 5D), indicating an increase in NAD^+^ consumption by the high glycolytic activity when mitochondrial respiration was suppressed by expression of POLGdn. Inhibition of NOX by p22phox siRNA knockdown or by DPI caused an additional 40%–50% decrease in NAD^+^, with a corresponding increase in NADH (Figure 5D). Besides, cellular NADP^+^/NADPH ratio was significantly increased in cells with POLGdn expression (Figure 5E). The above data suggest that NOX might by important to maintain high glycolytic activity in the cells with mitochondrial defects by supplying additional NAD+ from oxidation of NADH. Indeed, the membrane-associated NOX preparation was able to oxidize either NADPH or NADH as the substrates, and the oxidase activity was decreased by p22phox gene knockdown (Figure 5F). These data might explain why p22phox knockdown could cause NAD^+^ level decrease in the Tet/on cells.

Because NOX generates ROS, which might then stabilize HIF-1α to stimulate glycolysis [25], we tested if altering NOX activity would cause changes in ROS and HIF-1α. We knocked down p22phox using siRNA and then induced POLGdn expression for 4 d. Cellular O_2_^−^ and mitochondrial O_2_^−^ levels did not show any change on day 4 (Figure S2E–F), while knockdown p22phox causes a detectable decrease in cellular H_2_O_2_ (Figure S2G), consistent with the role of NOX in generating ROS outside the mitochondria. HIF-1α was not induced in the POLGdn cells even after 2 wk of Tet/on, while hypoxia induced a significant increase of HIF-1α in the same cells (Figure 5G). These data together suggest that induction of mitochondrial dysfunction did not significantly change HIF-1α expression. Thus, HIF-1α seemed not to play a major role in the POLGdn Tet/on cells to promote glycolysis.

### Cancer Cells with Compromised Mitochondrial Function Due to p53 Loss Show Elevated NOX Activity and Increased Sensitivity to NOX Inhibition

**Figure 6:**
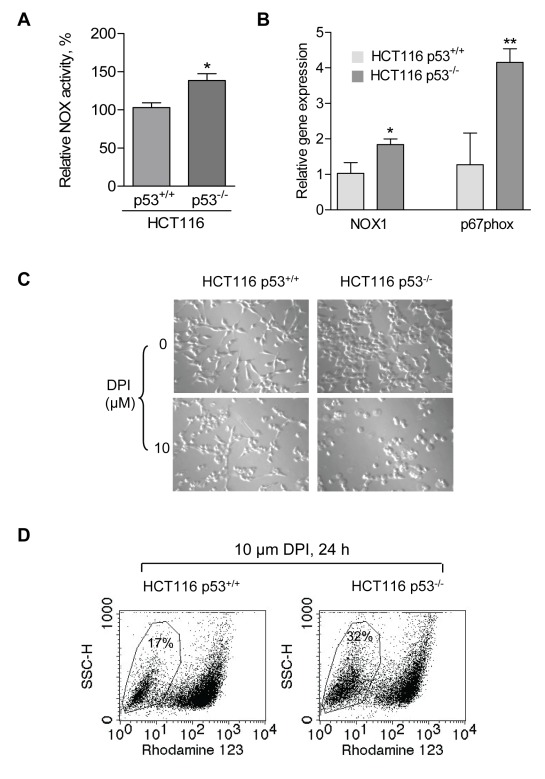
Cancer cells with loss of p53 and compromised mitochondrial respiration exhibit increased NOX and are sensitive to NOX inhibition. (A) Comparison of membrane-associated NOX activity in human colon cancer HCT116 cells with wild-type p53 (p53^+/+^) or complete loss of p53 (p53^−/−^). Error bars, ±SD. * *p*<0.05 (*n* = 3). (B) Increased gene expression of NOX components NOX1 and p67phox in HCT116 p53^−/−^ cells detected by qRT-PCR assay. Error bars, ±SD. * *p*<0.05; ** *p*<0.01 (*n* = 3). (C) Comparison of cell morphology and cell growth in HCT116 p53^+/+^ and HCT116 p53^−/−^ cells treated with the NOX inhibitor DPI (10 µM, 24 h). (D) Inhibition of NOX by DPI (10 µM, 24 h) preferentially induced loss of mitochondrial transmembrane potential in HCT116 p53^−/−^ cells.

It is known that the tumor suppressor p53 plays an important role in maintaining mitochondrial function through transcriptional activation of SCO2 and the loss of p53 leads to a decrease in mitochondrial respiration and increase in lactate generation [26],[27]. We reasoned that if NOX were important for the survival of cells with mitochondrial respiratory defect, the p53-null cells would be expected to have elevated NOX activity and be sensitive to NOX inhibition. To test this possibility, we compared NOX activity in human colon cancer cells (HCT116) with wild-type p53 or p53^−/−^. As shown in Figure 6A, the p53^−/−^ cells had a higher membrane-associated NOX activity. Further analysis by semi-quantitative RT-PCR revealed that the expression of the NOX components NOX1 and p67phox were increased in HCT116 p53^−/−^ cells (Figure S3A). Quantitative analysis using qRT-PCR showed that NOX1 and p67phox in HCT116 p53^−/−^ cells have about 2- and 4-folds increase in gene expression, respectively, compared to HCT116 p53^+/+^ cells (Figure 6B).

To evaluate the importance of increased NOX for the survival of HCT116 p53^−/−^ cells, we tested their sensitivity to NOX inhibitor DPI in comparison with the HCT116 p53^+/+^ cells. As shown in Figure 6C, after DPI treatment for 24 h, HCT116 p53^−/−^ cells exhibited a round-up morphology and detached, leading to a decrease in the number of viable cells when compared with HCT116 p53^+/+^ cells. To further confirm this different sensitivity, the effect of DPI on mitochondrial transmembrane potential was compared in both cell lines by flow cytometry using rhodamin-123 staining. Substantially more HCT116 p53^−/−^ cells (32%) lost their mitochondrial transmembrane potential after DPI treatment for 24 h, compared to 17% observed in the HCT116 p53^+/+^ cells (Figure 6D). These data suggest that NOX was up-regulated in cancer cells with loss of p53 and that inhibition of NOX could be a therapeutic strategy to preferentially kill these cancer cells. Indeed, cell death measured by annexin-V/PI analysis showed that the HCT116 p53^−/−^ cells were more vulnerable to DPI than the HCT116 p53^+/+^ cells (Figure S3B). To further confirm that p53^−/−^ cells have higher NOX activity, H1299 cells were transfected with p53wt expression plasmid to generate p53^wt^ stably expressed H1299-p53^wt^ cell line (Figure S3C). We observed that forced expression of wild-type p53 in H1299 cells have significantly decreased NOX activity (*p*<0.001) (Figure S3D).

### Upregulation of NOX in K-ras^G12V^-Expressing Cells and in Pancreatic Cancer Specimens

**Figure 7:**
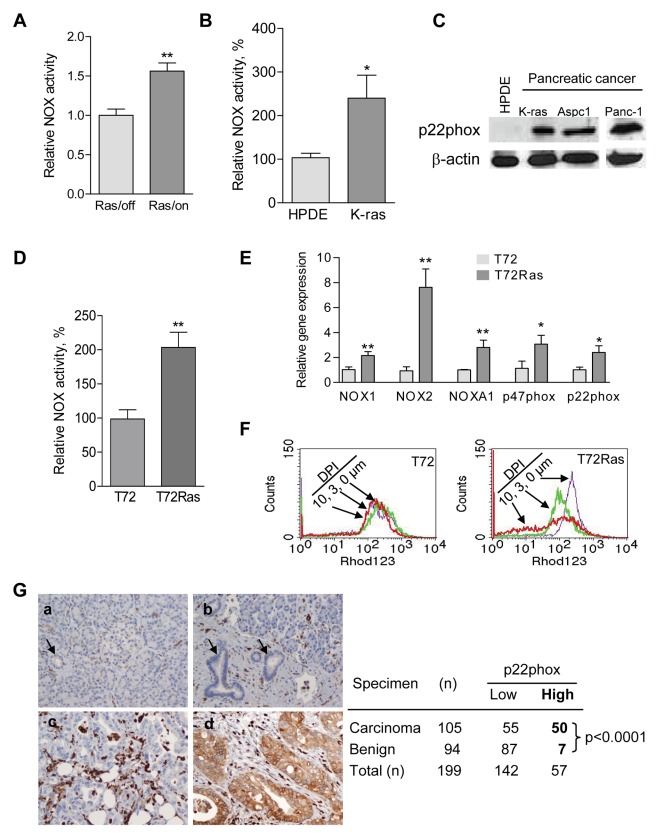
Elevation of NOX in Ras transformed cells and in primary pancreatic cancer tissues. (A) Increase of NOX activity in T-Rex 293 Tet/on cells with 1 mo K-ras^G12V^ induction compared with control. Error bars, ±SD. ** *p*<0.01 (*n* = 3). (B) NOX activity was substantially elevated in pancreatic K-ras^G12V^ stably transformed HPDE-kRas^G12V^ cells compared with the parental HPDE (human pancreatic ductal epithelial) cells. Error bars, ±SD. * *p*<0.05 (*n* = 3). (C) Increased protein level of p22phox in HPDE-K-ras^G12V^ cells and primary pancreatic cancer cells Aspc1 and Panc-1 compared to HPDE cells. (D) Increased NOX activity in the H-RAS^V12^-transformed (T72Ras) cells compared to normal ovarian epithelial cells (T72). Error bars, ±SD. ** *p*<0.01 (*n* = 3). (E) Increased gene expression of NOX components (NOX1, NOX2, NOXA1, p22phox, and p47phox) in the H-RAS^V12^-transformed T72Ras cells when compared to the parental T72 cells. Expression of mRNA was measured by qRT-PCR analysis. Error bars, ±SD. * *p*<0.05; ** *p*<0.01 (*n* = 3). (F) Preferential disruption of mitochondrial transmembrane potential by DPI (3–10 µM, 20 h) in H-RAS^V12^-transformed T72Ras cells compared with the parental T72 cells. Mitochondrial transmembrane potential was measured by flow cytometry using rhodamine-123 as a probe. (G) Representative tissue staining showing no expression of p22phox protein in normal pancreas (a, single arrow, normal pancreatic duct; double arrows, islet cells) and chronic pancreatitis (b, arrows, benign pancreatic ducts), and a moderately differentiated pancreatic ductal carcinoma (c) and strong positive staining in a moderately differentiated pancreatic ductal carcinoma (d). The strong positive staining in the inflammatory cells served as internal positive controls for our immunohistochemical stain (original magnification, 200×). Expression of p22phox in stage II pancreatic ductal carcinoma (PDC) and benign pancreatic tissue on microarray. p22phox expression is considered to be significantly different between PDC and benign group and higher in PDC group (*p*<0.0001 analyzed by Fisher's exact test).

Malignant transformation by oncogenic Ras is known to attenuate mitochondrial function and promote glycolysis [28],[29]. We introduced an inducible K-ras^G12V^ expression vector into the T-Rex 293 cells and observed that induction of K-ras^G12V^ expression caused a 50% decrease in mitochondrial respiration [30]. Importantly, this also caused a significant increase in NOX enzyme activity (Figure 7A) and elevated gene expression of the NOX components (Figure S4A). In a separate experiment, stable transformation of human pancreatic ductal epithelial (HPDE) cells with K-ras^G12V^ also induced NOX upregulation (Figure 7B and Figure S4B). Western blot analysis of the K-ras^G12V^ stably transformed cells and two naturally occurring pancreatic cancer cell lines (AsPC1 and Panc-1) showed that the protein level of p22phox, an essential component of the NOX enzyme complex, was substantially higher in pancreatic cancer cells than in non-malignant HPDE cells (Figure 7C). Using the *H-Ras^V12^*-transformed human ovarian epithelial cell pair [31],[32], we also found that NOX activity in *H-Ras^V12^*-transformed cells (T72Ras) was significantly higher than that in their non-tumorigenic parental T72 cells (Figure 7D). The increase in expression of *NOX1*, *NOX2*, *p22phox*, and *p47phox* in the H-Ras^V12^-transformed cells was demonstrated by semi-quantitative RT-PCR analysis (Figure S4C) and quantitatively confirmed by qRT-PCR (Figure 7E). To test if the elevated NOX activity is important for the survival of the *H-Ras^V12^*-transformed cells, we compared the sensitivity of T72Ras cells and the parental T72 cells to DPI. The transformed T72Ras cells were more vulnerable to NOX inhibition by DPI, leading to a substantial decrease of mitochondrial transmembrane potential (Figure 7F).

To test the clinical relevance of the above observations, we analyzed the expression of p22phox in clinical specimens using primary pancreatic tissue microarrays containing 105 cases of stage II pancreatic ductal carcinoma samples and 94 benign pancreatic tissues (normal and pancreatitis tissues). As shown in Figure 7G, about 48% (50/105) of the pancreatic cancer tissues exhibited a high protein level of p22phox, whereas only about 7% (7/94) of the benign pancreatic tissues were positive for p22phox. These data suggest that p22phox was significantly higher in pancreatic carcinoma than in non-malignant tissues (*p*<0.0001, Fisher's exact test).

### Suppression of NOX Exhibits Significant Inhibitory Effect against Pancreatic Cancer in vivo

**Figure 8:**
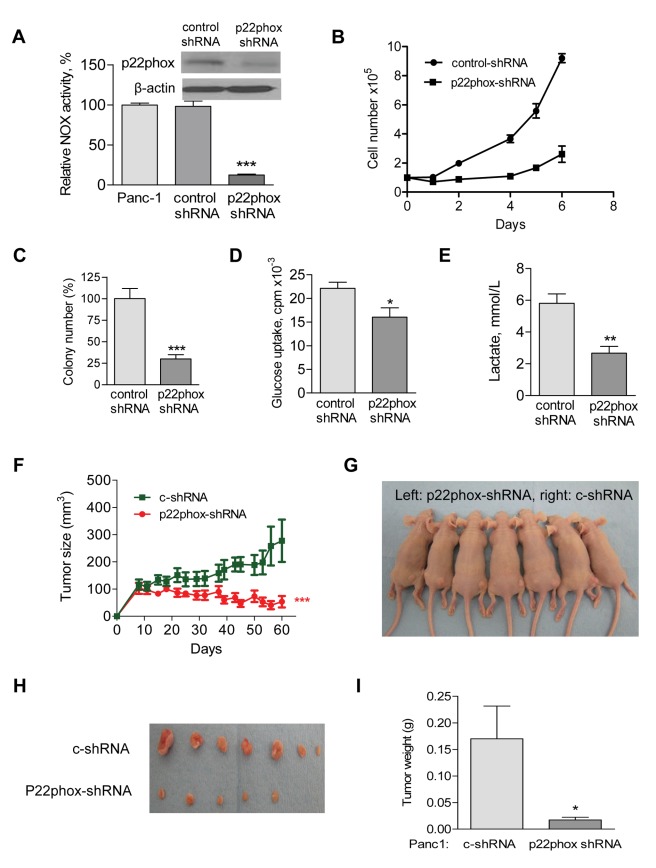
Inhibition of pancreatic tumor growth by NOX inhibition. (A) Decrease of NOX activity by stable shRNA knockdown of p22phox (p22phox-shRNA) in Panc-1 cells, but not in cells with control shRNA (c-shRNA) knockdown. *** *p*<0.001 (*n* = 3). Insert, Panc-1 cells were infected with p22phox-shRNA lentiviral particles, and the knockdown efficiency was detected by anti-p22phox antibody using Western blot. Non-targeting control shRNA lentiviral particles (c-shRNA) were used as negative control. (B) p22phox knockdown significantly decreased Panc-1 cell growth. 1×10^5^ cells were seed to six-well plates. Cell numbers were counted using Z2 coulter counter (Beckman Coulter) during 6 d of culture. (C) p22phox knockdown significantly suppressed colony formation of Panc-1 cells. 1×10^4^ cells were seed in 0.35% of the soft agar and assayed for colony formation after 2 wk. The numbers of colonies formed on soft agar were counted. Error bars, ±SD. *** *p*<0.001 (*n* = 3). (D) p22phox knockdown decreased glucose uptake in Panc-1 cells. Cells (1×10^6^) were incubated in 5 ml glucose-free DMEM medium for 4 h, followed by incubation with 0.2 µCi/mL ^3^H-2-deoxyglucose for 1 h. Cellular uptake of ^3^H-2-deoxyglucose was determined by liquid scintillation counting after the cells were washed 2 times with PBS and normalized by cell number. Error bars, ±SD. * *p*<0.05 (*n* = 3). (E) p22phox knockdown decreased lactate generation in Panc-1 cells. Lactate was measured 24 h after changing to fresh culture medium and normalized by cell number. Error bars, ±SD. ** *p*<0.01 (*n* = 3). (F–I) Each side of athymic nude mice (*n* = 7) received subcutaneously injections of 5×10^6^ Panc-1 cells bearing p22phox-shRNA (left flank) or c-shRNA (right flank). The mice were monitored for tumor growth and body weight throughout the experiment. All the mice were sacrificed when tumor size reached about 10% of body weight as mandated by the IACUC protocol. Tumor volume was calculated using the following equation: tumor volume (mm^3^) = L * W *(L+W)/2 * 0.526. (F) The tumor sizes were measured throughout the experiment to evaluate p22phox knockdown effect. Data represent tumor volume: mm^3^±SEM. *** *p*<0.001 (*n* = 7). (G) Photographs of athymic nude mice bearing p22phox-shRNA (left flank) or c-shRNA (right flank) xenografts. (H) Photograph and comparison of excised tumor size. (I) Tumor weight derived from p22phox-shRNA knockdown or c-shRNA knockdown was measured. Error bars, ±SEM. *p*<0.05 (*n* = 7).

To test the role of NOX in cancer cell survival, we stably knocked down p22phox expression in human pancreatic cancer cells (Panc-1), using p22phox shRNA lentiviral particles (Santa Cruz). The p22phox protein was substantially decreased and NOX activity was significantly decreased (Figure 8A) in p22phox-shRNA stably expressed cells compared with Panc-1 cells transduced with the control shRNA lentiviral particals. The knockdown of p22phox led to a significant decrease in cell growth (Figure 8B) and colony formation capacity (Figure 8C). Suppression of NOX expression also significantly decreased glucose uptake and lactate generation in pancreatic cancer cells (Figure 8D and 8E). To test the effect of NOX suppression on tumor growth in vivo, we subcutaneously inoculated the Panc-1 cells bearing p22phox-shRNA into the left flank of athymic nude mice (*n* = 7) and Panc-1 cells bearing control shRNA into the right flank of the same 7 mice (5×10^6^ cells/injection site). Suppression of p22phox expression significantly impaired tumor growth in vivo (Figure 8F and 8G). The average tumor volume in the p22phox-shRNA group was 53.4±20.9 mm^3^, compared to 277.6±77.8 mm^3^ in the control group. After 60 d of cell inoculation, the average tumor weight of the p22phox-shRNA xenografts was 17±5 mg, compared to 170±62 mg in the control group (Figure 8H and 8I). These data together suggest that NOX are essential for tumor growth in vivo.

We then used DPI, a chemical inhibitor of NOX [23], to test its potential therapeutic activity against pancreatic cancer in vivo. Athymic nude mice were inoculated with Panc-1 cells subcutaneously (Text S1). When the tumor grew to 100 mm^3^, the mice were divided into two groups for treatment with vehicle (PBS) as control or with DPI (1.5 mg/kg mouse, i.v., 5 times/week). Such treatment did not cause significant toxicity in the mice and there was no loss of body weight in the DPI-treated group (Figure S5A). The tumors grew progressively in the control group, whereas the DPI-treated group exhibited significant retardation in tumor growth (tumor volume 1,068±309.7 mm^3^ versus 139±80.4 mm^3^, Figure S5B). Some of the tumors in the treatment group showed complete regression (Figure S5C). The average tumor weights of the control group were significantly increased after about 10 wk compared with the DPI-treated group (478±98 mg versus 84±52 mg, *p*<0.01, Figure S5D). These data suggest that DPI could effectively inhibit pancreatic tumor growth without apparent toxic side effects.

## Discussion

In this study, we established a dominant-negative mitochondrial DNA polymerase γ inducible cell system, which enabled us to investigate the relationship between mitochondrial respiratory defect and metabolic alterations. Unlike ρ° cells derived by chronic exposure of cells to ethidium bromide that could also damage nuclear DNA, the POLGdn inducible cells provide a clean isogenic model system that allowed the induction of mitochondrial respiration suppression under well-defined conditions without causing direct nuclear DNA damage. This model made it possible to monitor metabolic alterations during the shift from oxidative respiration to high glycolysis and to examine the biochemical mechanisms in detail. The use of this cell system in the current study led to a novel finding that NOX was consistently up-regulated when mitochondrial respiration was suppressed by the expression of POLGdn, and this was further confirmed in cancer cells with loss of p53 or expression of oncogenic Ras. Further study showed that such NOX elevation is critical for the maintenance of high glycolytic activity in cells with mitochondrial respiratory defects. This conclusion is supported by multiple lines of evidence: (1) NOX expression and enzyme activity were elevated in cells with different degrees of mitochondrial respiration suppression induced by POLGdn, and in cancer cells with mitochondrial defect due to a loss of p53, or under the stress of Ras oncogenic signal. (2) In cells with mitochondrial respiratory defect, a suppression of NOX led to a decrease in NAD^+^ level, lower glucose uptake, and reduced ATP content, leading to loss of cell viability. (3) Knockdown of NOX enzyme component p22phox in Panc-1 cancer cells decreased glucose uptake and lactate generation, decreased cancer cell proliferation and colony formation capacity, and suppressed tumor growth in vivo.

Cells with mitochondrial respiration defect require a higher rate of glycolysis, and activation of NOX seems necessary to provide additional NAD^+^ to support the highly active glycolysis. Our study showed that suppression of NOX in these cells caused a decrease in NADH oxidation and lower cellular NAD^+^ level, a decrease in glycolysis and cellular ATP, and a loss of cell viability. Thus, NOX activation is important for providing additional NAD^+^ to support active glycolysis. This is a previously unrecognized function of NOX in energy metabolism. The production of NAD^+^ by lactate dehydrogenase (LDH) is traditionally thought to be the main pathway that maintains the supply of NAD^+^ for glycolysis. In normal cells with competent mitochondria and a moderate level of basal glycolytic activity, the NAD^+^ generated by LDH may be sufficient to support the glycolytic reaction catalyzed by GAPDH. However, in cells with mitochondrial dysfunction that require higher glycolytic activity, upregulation of NOX seems important to provide additional NAD^+^.

It is important to note that the POLGdn cells not only represent a model system to study mitochondrial respiration defect as shown in other ρ*°* cells, but it also allows us to investigate the metabolic changes with various degrees of mitochondrial dysfunction. The upregulation of NOX was observed not only at the stage when mitochondrial respiration was severely inhibited by POLGdn expressing, but we have also observed NOX activation in the early stage (day 2 of Tet/on) of mitochondrial dysfunction. NOX activation is also consistently observed in cancer cells with mitochondrial dysfunction, such as HCT116 colon cancer cells that lack p53 and thus have a disruption of cytochrome c oxidase complex (complex IV) in the mitochondria [26]. Synthesis of cytochrome c oxidase 2 (SCO2) is a transcriptional target of the tumor suppressor p53. The alterations of metabolic parameters including lactate generation and cellular ROS, NADH, and ATP levels observed in the HCT116 SCO2^−/−^ cell model [33] were similar to those seen in the POLGdn-Tet/on cells in this study. Interestingly, the Ras oncogene has recently been shown to cause mitochondrial dysfunction by disrupting mitochondrial complex activity [30],[34], which is consistent with our findings in this study. Stable transfection of human pancreatic ductal epithelial (HPDE) cells by K-ras^G12V^, induced expression of K-ras^G12V^ in T-Rex 293 Tet/on cells, or stable H-ras^V12^ transformation in human ovarian epithelial cells led to a significant increase in NOX enzyme activity. Moreover, the protein level of p22phox was found to be significantly elevated in human primary pancreatic cancer tissues. These observations together suggest that NOX up-regulation may be an important cellular adaptive response to mitochondrial dysfunction to enable the increase in glycolytic activity. As such, NOX upregulation may be significant in many pathological processes related to mitochondrial respiratory inhibition, especially in cancer cells where mitochondrial respiratory dysfunction and metabolic abnormalities are prominent.

The molecular mechanisms by which divergent triggers including POLGdn, oncogenic Ras, and loss of p53 cause upregulation of NOX remain unclear. In the study, we observed that the expression of various NOX family members was upregulated in cells with mitochondrial dysfunction. A recent study suggests that SIRT1, a NAD^+^-dependent histone deacetylase, is involved in the negative regulation of NOX1 expression [35]. It is possible that mitochondrial dysfunction induced by various factors would lead to a decrease in cellular NAD^+^ content due to active glycolysis that consumes NAD^+^, and therefore release the SIRT1 suppression on NOX1 expression. Further study is needed to investigate this possibility.

Conventionally, the membrane-bound NOX enzyme complex is considered as ROS-generating machinery in phagocytes involved in the defense against microorganisms and mediating certain inflammatory processes. Subsequently, nonphagocytic NOX family of proteins homologous to gp91phox and other subunits have been shown to generate ROS in nonphagocytic cells and have been thought to contribute to various cancer cell proliferation and progression [15],[16],[36],[37]. Our study revealed an important function of NOX in cellular energy metabolism, especially in cancer cells with mitochondrial dysfunction. The ability of NOX inhibition to suppress pancreatic cancer cell proliferation, to abrogate colony formation ability, and to significantly suppress tumor growth in vivo suggests the feasibility to target NOX for cancer treatment. Since pancreatic cancer is highly resistant to many anticancer agents currently used in clinic, targeting NOX may have significant clinical implications and merit further investigation.

## Materials and Methods

### Generation of Mitochondrial Respiration-Defective Model System

T-Rex 293 cells containing the pcDNA6/TR vector were obtained from Invitrogen. Full-length POLGdn cDNA harboring D1135A mutation and a FLAG-tag was excised by restriction enzyme EcoRI digestion from the POLGdn plasmid described previously [8]. The POLGdn fragment was subcloned into a mammalian expression vector pcDNA4/TO (Invitrogen) at EcoRI site and verified by nucleotide sequencing. The plasmid with correct orientation was verified by HindIII or XhoI enzyme digestion. The resulting POLGdn plasmid was then transfected into T-Rex 293 cells using lipofectamine 2000 (Invitrogen) to generate Tet/on inducible cell line. POLGdn positive colonies were selected by Zeocin (250 µg/ml) for 15 d, and expression of POLGdn was verified by 1 µg/ml doxycycline induction and Western blot analysis using anti-FLAG antibody (Sigma).

### Metabolic Measurements

Oxygen consumption, glucose uptake, and lactate generation were described previously [18]. For measurement of oxygen consumption, cells were trypsinized and resuspended in 1 ml fresh culture medium pre-equilibrated with 21% oxygen at 37°C followed by applying the cells to the sealed respiration chamber of a Clark-type oxygen measuring system (Oxytherm, Hansatech Instrument, Cambridge, United Kingdom) with constant stirring. To measure cellular glucose uptake, cells in exponential growth phase were washed with glucose-free medium and incubated in fresh glucose-free RPMI 1640 medium for 3 h followed by an incubation with 0.2 Ci/mL ^3^H-2-deoxyglucose for 1 h. The glucose uptake represented by ^3^H radioactivity was determined by liquid scintillation counting and normalized by cell number. To measure lactate production, cells in 80% confluent were replenished with fresh medium. Aliquots of the medium were removed at the indicated time for measurement of lactate, using an Accutrend lactate analyzer (Roche, Mannheim, Germany). At each time point, cell number was also counted for normalization of lactate generation. Cellular ATP contents were measured using CellTiter-Glo Luminescent Cell Viability Assay kit (Promega) according to manufacturer's recommendations.

### Flow Cytometry

Flow cytometry determination of ROS, mitochondrial membrane potential, and cell death was performed using a BD Biosciences FACSCalibur flow cytometer (Mountain View, CA) and analyzed using the CellQuest software (Becton Dickinson). Cellular superoxide level was measured by incubating cells with 200 ng/ml HET for 1 h and mitochondrial superoxide level was measured by incubating the cells with 5 µm MitoSox Red for 60 min. Intracellular H_2_O_2_ contents were measured by incubating cells with 4 µM DCF-DA at 37°C for 1 h and mitochondrial transmembrane potential was assessed by incubating cells with 1 µM Rhodamine-123 (Rho-123) for 30 min before flow cytometer analysis. Apoptosis was determined by using the annexin-V and PI double staining method.

### Assay for NAD(P)H Oxidase Activity and SOD1 Activity

NAD(P)H oxidase activity was measured by a lucigenin-derived chemiluminescence assay as described [21],[22]. Briefly, 5–7 µg homogenized protein was incubated with its substrate 100 µM NADH or NADPH in a phosphate buffer (50 mM, pH 7.0) containing 150 mM NaCl and 1 mM EGTA for 15 min, followed by an addition of 5 µM lucigenin for 15 min in dark. The chemiluminescent signal (photon emission) was measured using a Turner 20/20 luminometer (Turner Designs, Sunnyvale, CA). No activity could be measured in the absence of NADH or NADPH. Experiments were also performed with the following pharmacologic inhibitors: a flavoprotein inhibitor DPI, a NOS inhibitor L-NAME, a mitochondrial respiratory chain complex I inhibitor rotenone, or a xanthine oxidase inhibitor oxypurinol.

Superoxide dismutase 1 (SOD1) activity was assayed using a SOD assay kit (Cayman Chemicals) per the manufacturer's instructions. Briefly, cells were collected by centrifugation at 1,000× g for 10 min at 4°C, lysed, and centrifuged at 1,500× g for 5 min at 4°C. Cytosolic fraction containing SOD1 was obtained from further centrifugation at 10,000× g for 15 min.

### NAD^+^ and NADH Measurement

Intracellular NAD^+^ and NADH were quantified by HPLC as described previously [38] with some modifications. Briefly, after trypsinization, 6×10^6^ cells were suspended in DMEM medium without FBS and collected by centrifugation at 1,400 rpm for 5 min. Cells were then shock-frozen with liquid-nitrogen. The cell pellets were resuspended in 150 µl extraction buffer containing 7 volumes of ethanol and 3 volumes of 10 mM potassium phosphate buffer, pH 8.5. The cells were disrupted by sonication and then kept at room temperature for 30 min to release cellular contents. Lysates were cleared by centrifugation at 13,000 rpm at 4°C for 15 min. 100 µl of the supernatant were subjected to HPLC analysis using an anion-exchange column (Partisil-10 SAX, Whatman) at a flow rate of 1 ml/min from 100% buffer A (5 mM NaH_2_PO_4_, pH 4.0) to 100% buffer B (250 mM NAH_2_PO_4_+0.5 M NaCl, pH 4.75) over 20 min, followed by another 5 min isocratic 100% buffer A. Pure NAD^+^ and NADH were used as reference standards and for quantitative calibration. NAD^+^ and NADH were detected by UV absorbance at 260 nm and 340 nm with retention times of 8.9 min and 15.4 min, respectively. The peaks corresponding NAD^+^ and NADH were collected according to the standards' retention time, freeze-dried in a lyophilizor, and further confirmed by MALDI-MS analysis (Bruker Daltonics). In HPLC analysis, the amount of cellular NAD^+^ and NADH of each sample was quantified based on the peak area compared to the standard curve generated by NAD^+^ and NADH standards.

### Analysis of p22phox Expression in Pancreatic Tissues on Microarray

Immunohistochemical staining for p22phox was performed on 4-µm unstained sections from the tissue microarray blocks consisting of 105 stage II pancreatic ductal carcinoma and their paired non-neoplastic pancreatic tissue samples from patients who underwent pancreaticoduodenetomy at our institution. The use of clinical specimens for tissue array study was approved by the Institutional Review Board of MD Anderson Cancer Center. To retrieve the antigenicity, the tissue sections were treated at 100°C in a steamer containing 10 mmol citrate buffer (pH, 6.0) for 60 min. The sections were then immersed in methanol containing 0.3% hydrogen peroxidase for 20 min to block the endogenous peroxidase activity and were incubated in 2.5% blocking serum to reduce nonspecific binding. The sections were then incubated with a rabbit polyclonal antibody against p22phox (Santa Cruz, 1∶100 dilution) at 4°C overnight, washed, and then incubated with secondary antibody at room temperature for 60 min. Standard avidin-biotin immunohistochemical analysis of the sections was done according to the manufacturer's recommendations (Vector Laboratories, Burlingame, CA) and photographed using a digital camera attached to the microscope. The staining results were evaluated by a gastrointestinal pathologist. Since all the pancreatic cancer samples showed either negative or diffuse staining for p22phox, the levels of p22phox expression were graded based on the staining intensity as negative (0), weak (1), moderate (2), and strong (3). P22phox expression was categorized as p22phox-low (intensity score of 0 or 1) or p22phox-high (intensity score of 2 or 3).

### Assay of in vivo Antitumor Activity of NOX Inhibition by p22phox-shRNA

The experiments with mouse xenografts were carried out according to the protocols approved by the Institutional Animal Care and Use Committee (IACUC) of the University of Texas MD Anderson Cancer Center. Each side of the seven 10-wk-old athymic nude mice received a subcutaneous injection of 5×10^6^ Panc-1 cells bearing p22phox-shRNA (p22phox-shRNA, left flank) or control shRNA (c-shRNA, right flank). Tumor size and body weight were measured throughout the experiment. Moribund animals were sacrificed as mandated by the IACUC protocol, and the tumor weight was recorded. Tumor volume was calculated using the equation: tumor volume (mm^3^) = L * W *(L+W)/2 * 0.526.

### Statistical Analyses

The Kolmogorov-Smirnov test (Cell Quest Pro software, Becton-Dickinson, San Jose, CA, USA) was used to evaluate the significant difference between control and test samples in flow cytometry analysis. For comparison of the statistical differences of more than two groups, one-way ANOVA and Newman-Keul's multiple comparison test was used. All other statistical significant difference analyses were evaluated using Student's *t* test (Prism GraphPad, San Diego, CA). Statistical differences between p22phox expression in benign and malignant pancreatic tissue groups on microarray were evaluated by Fisher's exact test. A *p* value of less than 0.05 was considered statistically significant.

## Supporting Information

Figure S1 Figure S1 is related to Figure 4. (A) Comparison of mRNA expression of NOX family genes in Tet/off and Tet/on (day 8) cells, measured by semi-quantitative RT-PCR analysis. (B) Increase in NOXA1 and p47phox mRNA expression in the Tet/on cells, measured by semi-quantitative RT-PCR at the indicated time points after POLGdn expression. (C) Comparison of cell proliferation and morphology in cells with or without mitochondrial defect induced by POLGdn (Tet/on or Tet/off cells) treated with the NOX inhibitor DPI. The POLGdn-inducible T-Rex 293 cells were pre-incubated with or without doxycycline for 8 d and then treated with the indicated concentrations of DPI for 24 h. Cell density (as an indication of proliferation) and morphology were examined under a microscope.
(TIF)

Figure S2 Figure S2 is related to Figure 5. (A–B) siRNA knockdown of the NOX1 and p22phox in Tet/off or Tet/on cells (at day 4), respectively. Cells were transfected with siRNAs against NOX1, p22phox, or scramble RNA (SC) for 96 h. Knockdown efficiency was evaluated by qRT-PCR. (C–D) Mass spectrometry confirmation of NADH and NAD+ peaks from HPLC analysis. (C) Identification of NADH eluted at 15–15.5 min from SAX column in multiple salt forms. (D) Identification of NAD+ corresponding to the NAD+ peak eluted at 8.5–9.0 min. (E–G) Detection of cellular O_2_^−^, mitochondrial O_2_^−^, and cellular H_2_O_2_ in p22phox siRNA transfected Tet/on (d4) cells. Tet/off cells were used as control.
(TIF)

Figure S3 Figure S3 is related to Figure 6. (A) Increased expression of NOX components NOX1, NOX5, and p67phox in HCT116 p53−/− cells detected by semi-quantitative RT-PCR assay. (B) HCT116 p53−/− and HCT116 p53+/+ cells were seeded in six-well plates. Cells were cultured to subconfluent and then treated with 30 µM DPI for 42 h followed by annexin-V/PI double staining and flow cytometer analysis. The number in each panel indicates percentage of death cells. (C) H1299 cells were stably transfected with p53wt-pcDNA3.1/zeocin plasmid. p53wt expression in H1299 was confirmed by Western blot using anti-p53 antibody. (D) Comparison of membrane-associated NOX activity in H1299 cells and p53wt stably transfected H1299 cells (H1299-p53wt). Error bars, ±SD. *** *p*<0.001 (*n* = 3).
(TIF)

Figure S4 Figure S4 is related to Figure 7. (A) Real-time PCR revealed that mRNA expression of NOX2 and NOXA1 were upregulated after induction of K-Ras in T-Rex 293 cells. (B) NOX2 and NOXA1 expression were also upregulated in K-ras transformed pancreatic ductal epithelial cells (HPDE). Data are shown as mean ± SD of triplicate samples from two independent experiments. *p*<0.0001 compared to cells without K-ras induction. (C) Increased expression of NOX components NOX1, NOX2, p22phox, NOXA1, and p47phox in T72Ras cells detected by semi-quantitative RT-PCR assay.
(TIF)

Figure S5 Figure S5 is related to Figure 8. Ten 9-wk-old nude mice were inoculated subcutaneously with Panc-1 cells (5×10^6^/0.15 ml/mouse) and randomly divided into two groups (five mice each). After tumor formation (about 100 mm^3^), the treatment group received DPI (1.5 mg/kg mouse, i.p.) 5 times per week. The control group received an equal volume of PBS control. The mice were monitored for tumor growth and body weight throughout the experiment. (A) The body weights of the nude mice were measured during the drug treatment to estimate toxicity. Error bars, ±SEM (*n* = 5). (B) The tumor sizes were measured throughout the experiment to evaluate DPI effect. Data represent tumor volume: mm^3^±SEM (*n* = 5). (C) Photographs of nude mice bearing Panc-1 xenografts on day 65 of DPI treatment (one mouse from each group was sacrificed 2 d before). PBS treatment was used as control. (D) Tumor weight derived from control and DPI treated mice on day 65 after DPI treatment was measured. Error bars, ±SEM. *p*<0.01 and *n* = 5.
(TIF)

Text S1 Supplemental materials and methods and supplemental references.
(DOC)

## Abbreviations

COII: mtDNA-encoded cytochrome c oxidase subunit II
DPI: diphenyleneiodonium
HET: hydroethidine
HKII: hexokinase II
HPDE: human pancreatic ductal epithelial cells
L-NAME: Nω-nitro-L-arginine mrthyl ester hydrochloride
mtDNA: mitochondrial DNA
NOX: NAD(P)H oxidase
NOXA1: NOX activator 1
NOXO1: NOX organizer 1
POLG: mitochondrion DNA polymerase gamma
POLGdn: dominant negative form of POLG
ROS: reactive oxygen species
SOD1: superoxide dismutase 1
SOD2: superoxide dismutase 2
