# Correction: Novel Role of NOX in Supporting Aerobic Glycolysis in Cancer Cells with Mitochondrial Dysfunction and as a Potential Target for Cancer Therapy

**DOI:** 10.1371/journal.pbio.1002616

**Published:** 2017-12-11

**Authors:** Weiqin Lu, Yumin Hu, Gang Chen, Zhao Chen, Hui Zhang, Feng Wang, Li Feng, Helene Pelicano, Hua Wang, Michael J. Keating, Jinsong Liu, Wallace McKeehan, Huamin Wang, Yongde Luo, Peng Huang

The authors would like to clarify several issues recently raised by the *PLOS Biology* editors.

In Fig 1 and Fig 2 the same image of beta-actin loading control has been mistakenly used for panels Fig 1B, Fig 1E and Fig 2D. Please see the corrected figures here, in which the beta-actin control for Fig 1E and Fig 2D is replaced with the correct image. The figure legends remain unchanged.

**Fig 1:**
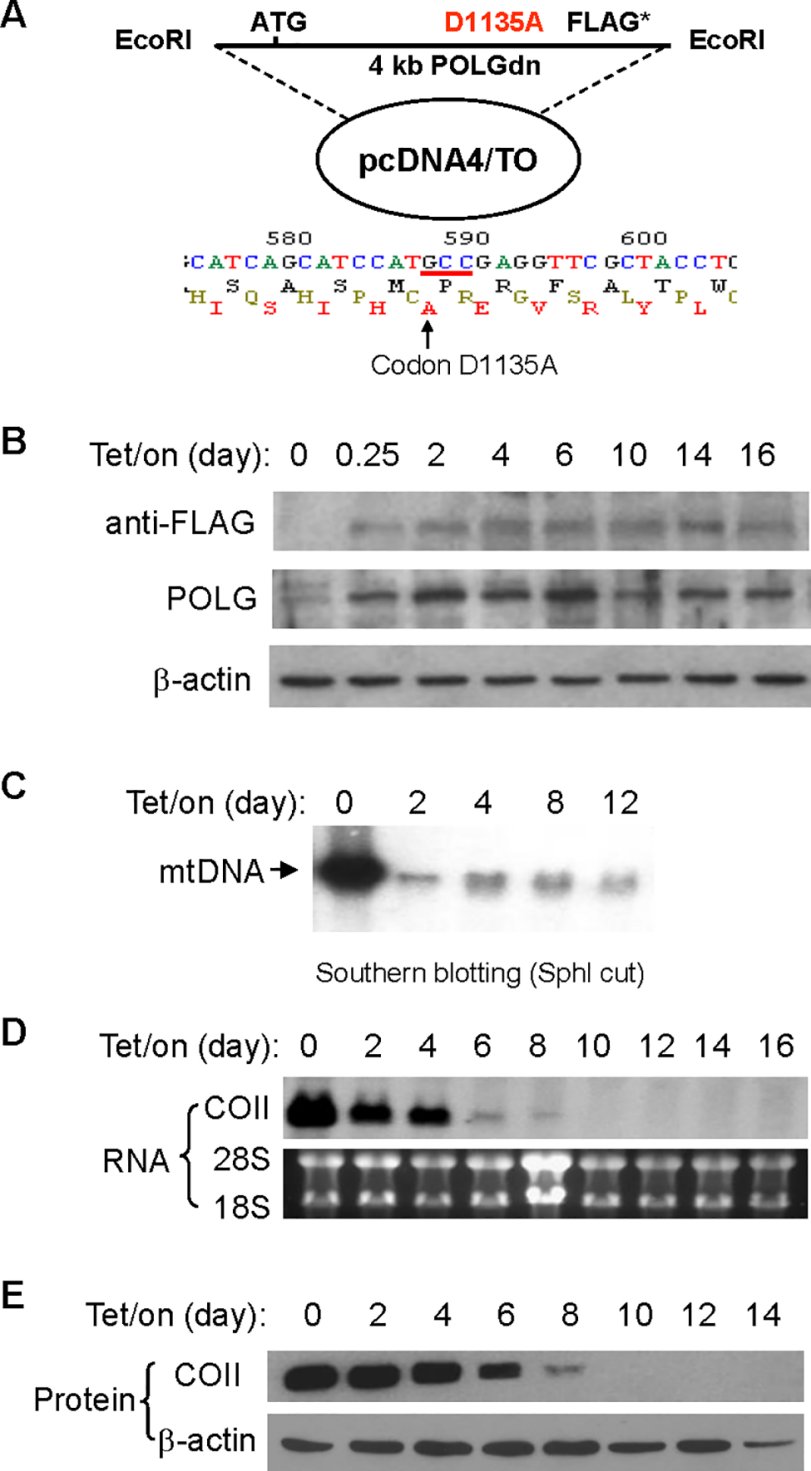
POLGdn expression led to the depletion of mtDNA-encoded respiratory chain components. (A) POLGdn-pcDNA4/TO construct and nucleotide sequencing analysis confirming the D1135A mutation. (B) Induction of POLGdn expression by doxycycline. T-Rex293 cells carrying POLGdn construction were incubated with doxycycline at an indicated time point. POLGdn expression was detected by anti-FLAG antibody, while both the endogenous POLG and POLGdn proteins were detected by anti-POLG antibody using Western blot assay. (C) Dramatic decrease of mtDNA by expression of POLGdn. Southern blot assay was used to measure mtDNA content. 10 µg total cellular DNA (including genomic DNA and mtDNA) from each sample was digested with SphI to linealize the circular mtDNA, followed by gel electrophoresis. ^32^P-labeled mitochondrial COII DNA fragment was used as a probe to detect mtDNA. (D) Assay of mtDNA-encoded COII RNA expression by northern blot analysis. (E) Detection of mitochondrial DNA-encoded COII protein by Western blot assay.

**Fig 2:**
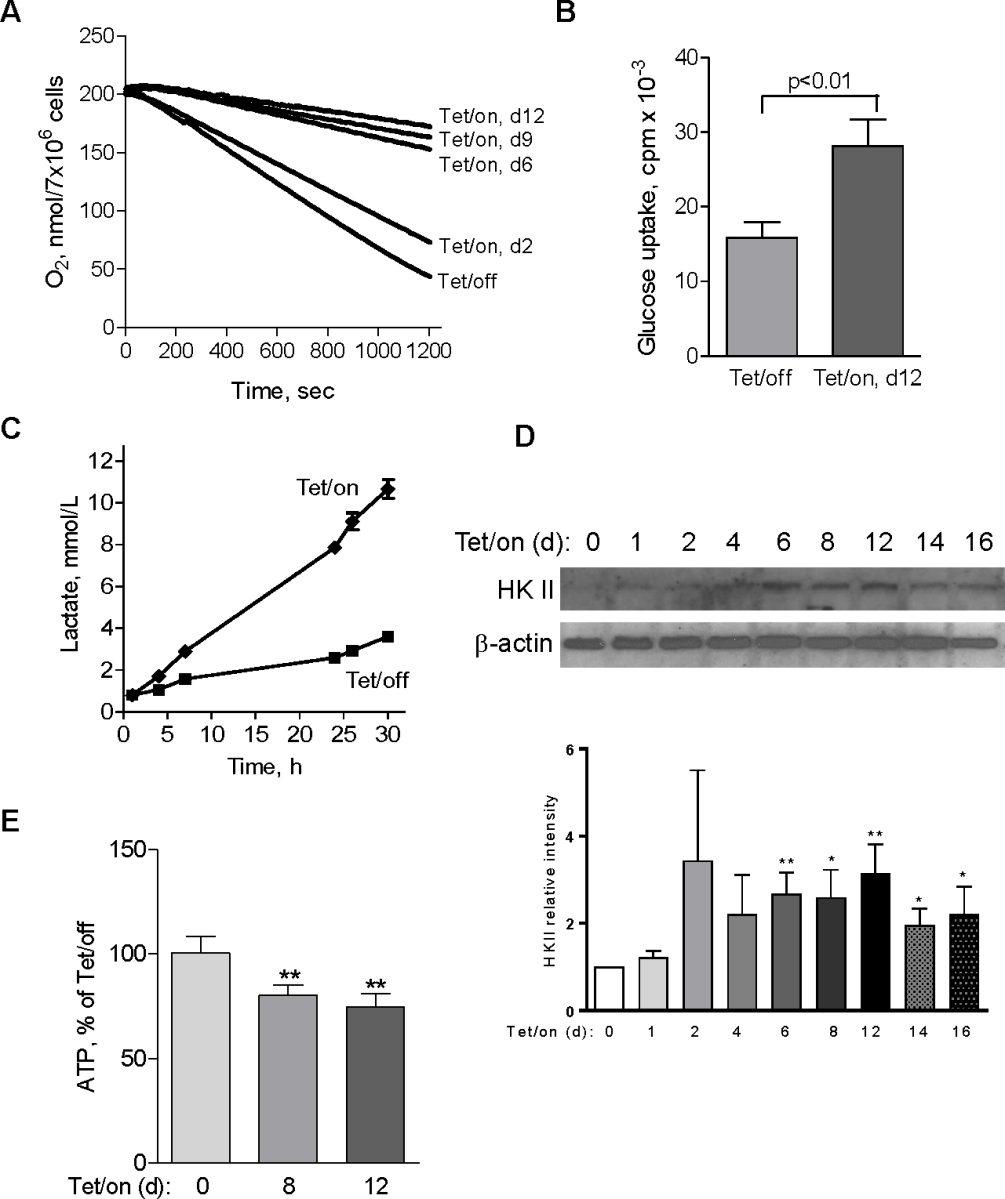
Suppression of mitochondrial respiration by POLGdn expression led to an elevation of glycolysis. (A) Time-dependent decrease in cellular oxygen consumption following POLGdn expression. Reduction of oxygen consumption was observed as early as 2 d after POLGdn expression, and the cells dramatically decreased their ability to consume oxygen with prolonged POLGdn expression. (B) Increased glucose uptake in POLGdn-expressing cells (Tet/on, d12). Cells (2×10^6^) were incubated in 5 ml glucose-free RPMI1640 medium for 2 h, followed by incubation with 0.2 μCi/mL ^3^H-2-deoxyglucose for 1 h. Cellular uptake of 3H-2-deoxyglucose was determined by liquid scintillation counting after the cells were washed two times with PBS. Error bars, ±SD. p<0.01 (n = 3). (C) Increased lactate generation in Tet/on cells. Lactate in Tet/off and Tet/on (day 12) cells was measured at the indicated time points after changing to fresh culture medium. (D) Increased protein level of hexokinase II (HKII) following POLGdn expression. Upper panels show representative HKII protein by Western blotting assay at the indicated days after POLGdn induction by doxycycline. Lower panels show quantification of Western blot results using scanning and ImageJ software. Results are expressed as integrated optical density. Each sample was normalized to β-actin content. Each bar represents the mean ± SEM of three independent experiments. * p<0.05; ** p<0.01. (E) Comparison of cellular ATP levels in cells with or without POLGdn expression. Cellular ATP contents in Tet/on cells (days 8 and 12) were measured and compared with the Tet/off cells. Error bars, ±SD (** p<0.01 and n = 3).

In Fig 2D, two time points (days 1 and 14) are not included. As requested by the editors, the authors have re-run this experiment, using the same cell lines as used in the original experiments and, with this newly generated data, provided a corrected version of the figure here that includes both days 1 and 14. In addition, all three gel images from replicate experiments for Fig 2D are presented in a new S1 File. Quantitation of these three replicate gels is presented as a bar graph in revised Fig 2D. The editors have verified the data underlying the corrected panel and are satisfied that these continue to uphold the conclusions from this figure.

## Supporting information

S1 File Replicate gels of Fig 2D. POLGdn cells were induced by doxycycline for various times (Tet/on, 1–16 days) as indicated. The cell lysates were then analyzed for expression of HKII protein by western blotting. β-actin was also blotted as a protein loading control. For quantitative analysis, the band density for each time point was first divided by the band density of the Tet/off control band (0 day) and expressed as a relative value (fold). The value represented by each HKII band was then further normalized by the corresponding β-actin band value. The detailed quantitation data were shown in Supplemental Table 1 on the next page.
(PDF)
