# Supplementary figures and images for: Novel Role of NOX in Supporting Aerobic Glycolysis in Cancer Cells with Mitochondrial Dysfunction and as a Potential Target for Cancer Therapy
Source: PLoS Biol. 2012 May 8;10(5):e1001326. doi: 10.1371/journal.pbio.1001326 (PMC3348157; doi:10.1371/journal.pbio.1001326)

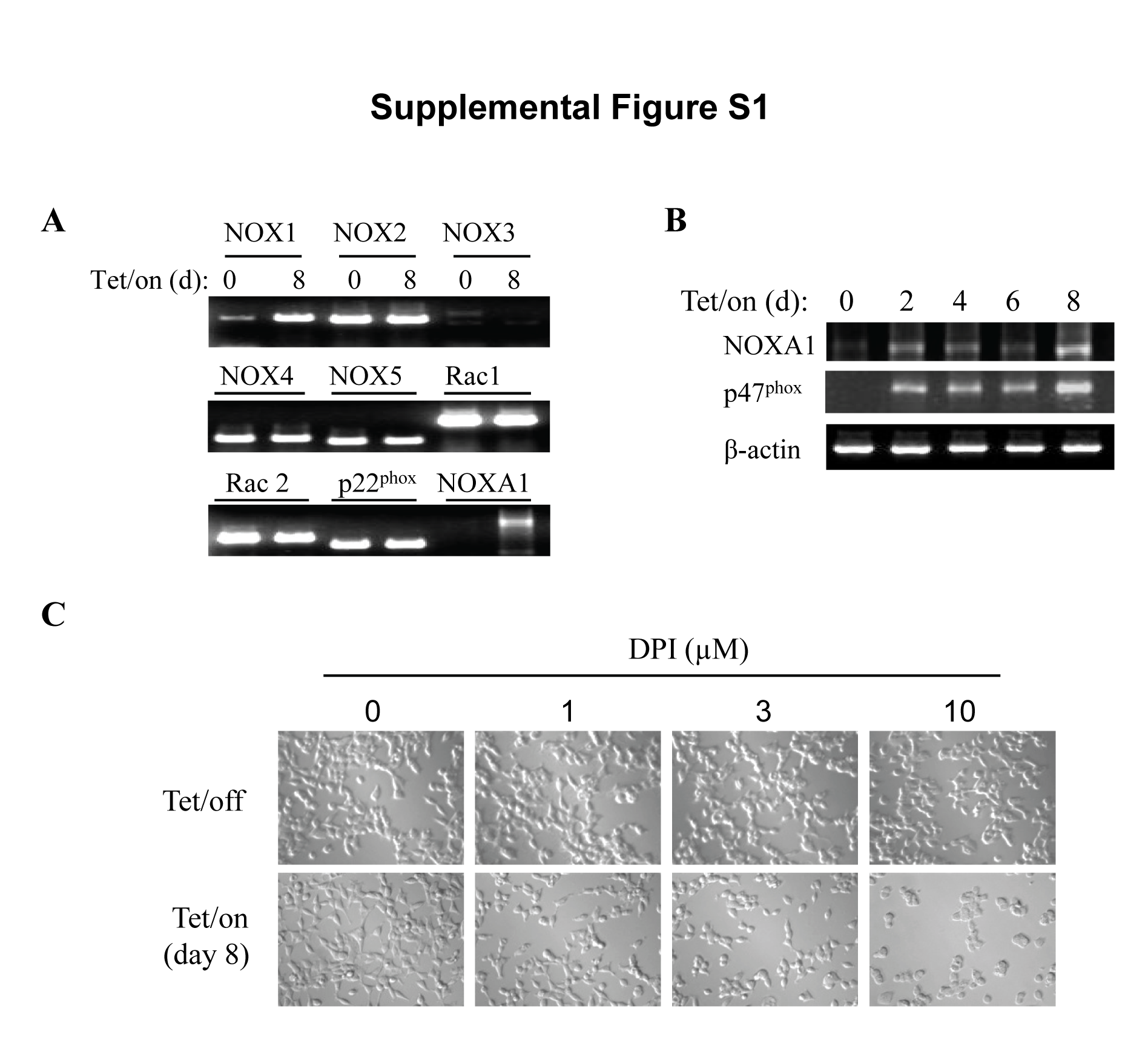

Supplement: Figure S1 — is related to Figure 4. (A) Comparison of mRNA expression of NOX family genes in Tet/off and Tet/on (day 8) cells, measured by semi-quantitative RT-PCR analysis. (B) Increase in NOXA1 and p47phox mRNA expression in the Tet/on cells, measured by semi-quantitative RT-PCR at the indicated time points after POLGdn expression. (C) Comparison of cell proliferation and morphology in cells with or without mitochondrial defect induced by POLGdn (Tet/on or Tet/off cells) treated with the NOX inhibitor DPI. The POLGdn-inducible T-Rex 293 cells were pre-incubated with or without doxycycline for 8 d and then treated with the indicated concentrations of DPI for 24 h. Cell density (as an indication of proliferation) and morphology were examined under a microscope. (TIF) [file pbio.1001326.s001.tif]

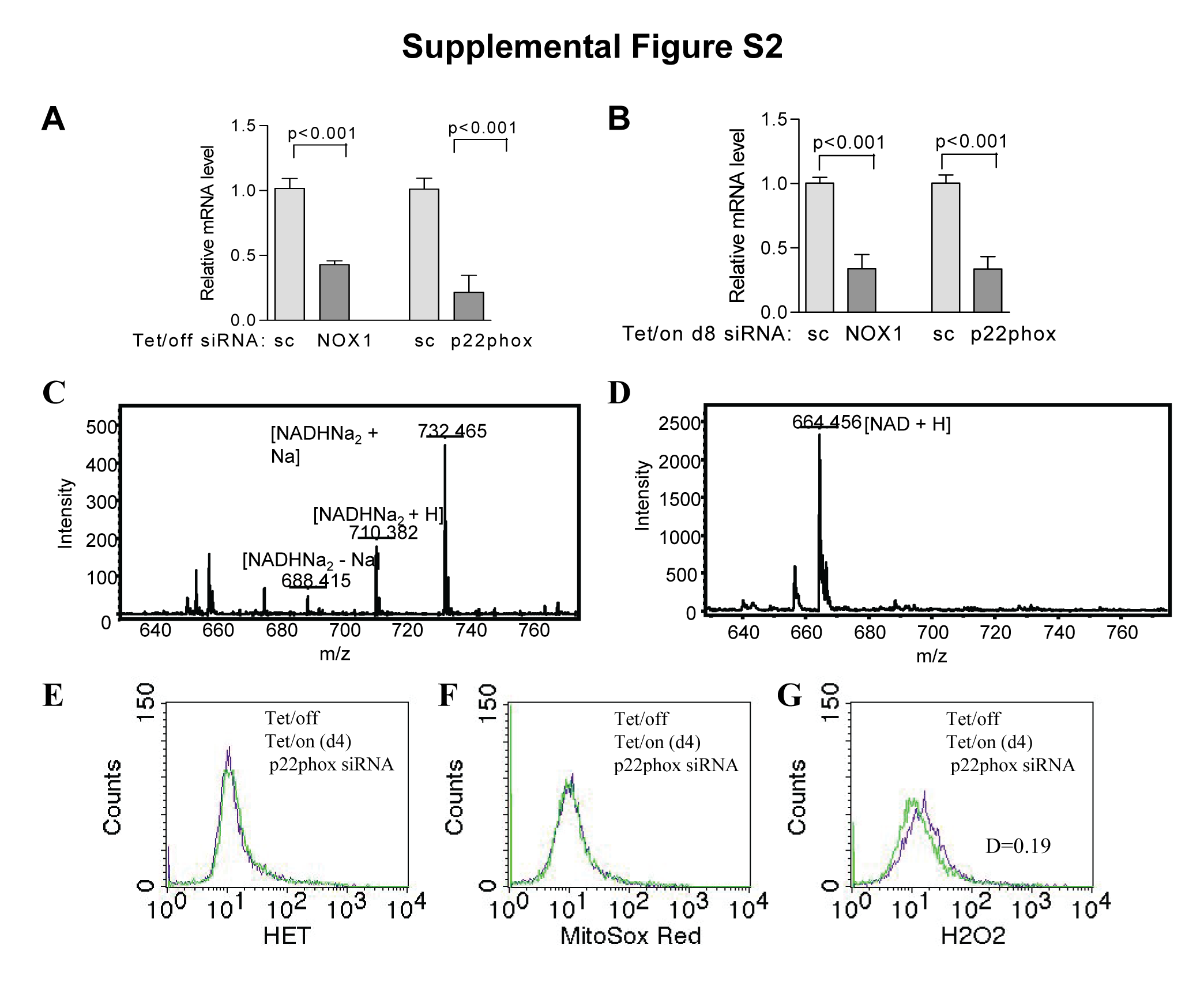

Supplement: Figure S2 — is related to Figure 5. (A–B) siRNA knockdown of the NOX1 and p22phox in Tet/off or Tet/on cells (at day 4), respectively. Cells were transfected with siRNAs against NOX1, p22phox, or scramble RNA (SC) for 96 h. Knockdown efficiency was evaluated by qRT-PCR. (C–D) Mass spectrometry confirmation of NADH and NAD+ peaks from HPLC analysis. (C) Identification of NADH eluted at 15–15.5 min from SAX column in multiple salt forms. (D) Identification of NAD+ corresponding to the NAD+ peak eluted at 8.5–9.0 min. (E–G) Detection of cellular O2 −, mitochondrial O2 −, and cellular H2O2 in p22phox siRNA transfected Tet/on (d4) cells. Tet/off cells were used as control. (TIF) [file pbio.1001326.s002.tif]

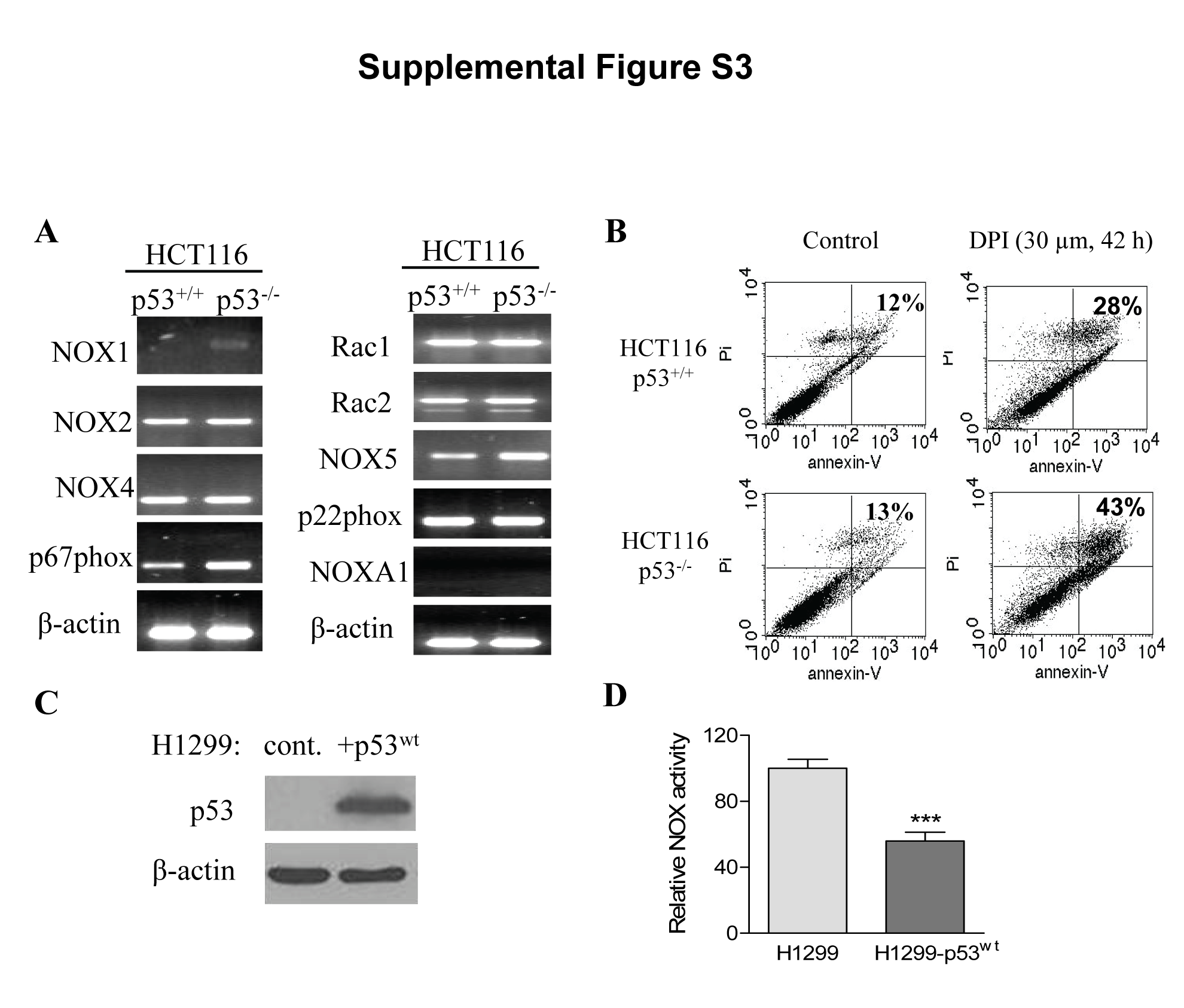

Supplement: Figure S3 — is related to Figure 6. (A) Increased expression of NOX components NOX1, NOX5, and p67phox in HCT116 p53−/− cells detected by semi-quantitative RT-PCR assay. (B) HCT116 p53−/− and HCT116 p53+/+ cells were seeded in six-well plates. Cells were cultured to subconfluent and then treated with 30 µM DPI for 42 h followed by annexin-V/PI double staining and flow cytometer analysis. The number in each panel indicates percentage of death cells. (C) H1299 cells were stably transfected with p53wt-pcDNA3.1/zeocin plasmid. p53wt expression in H1299 was confirmed by Western blot using anti-p53 antibody. (D) Comparison of membrane-associated NOX activity in H1299 cells and p53wt stably transfected H1299 cells (H1299-p53wt). Error bars, ±SD. *** p<0.001 (n = 3). (TIF) [file pbio.1001326.s003.tif]

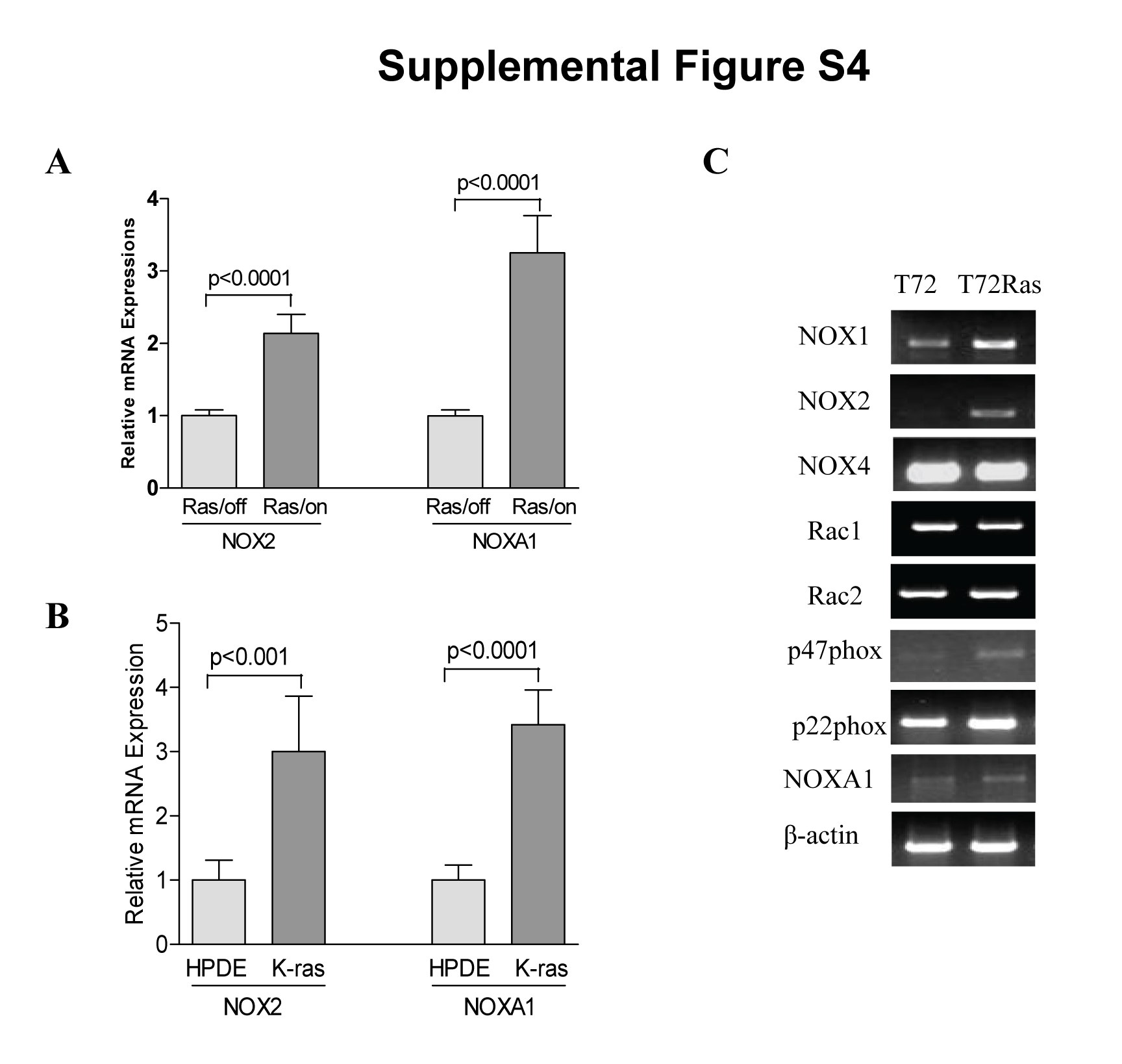

Supplement: Figure S4 — is related to Figure 7. (A) Real-time PCR revealed that mRNA expression of NOX2 and NOXA1 were upregulated after induction of K-Ras in T-Rex 293 cells. (B) NOX2 and NOXA1 expression were also upregulated in K-ras transformed pancreatic ductal epithelial cells (HPDE). Data are shown as mean ± SD of triplicate samples from two independent experiments. p<0.0001 compared to cells without K-ras induction. (C) Increased expression of NOX components NOX1, NOX2, p22phox, NOXA1, and p47phox in T72Ras cells detected by semi-quantitative RT-PCR assay. (TIF) [file pbio.1001326.s004.tif]

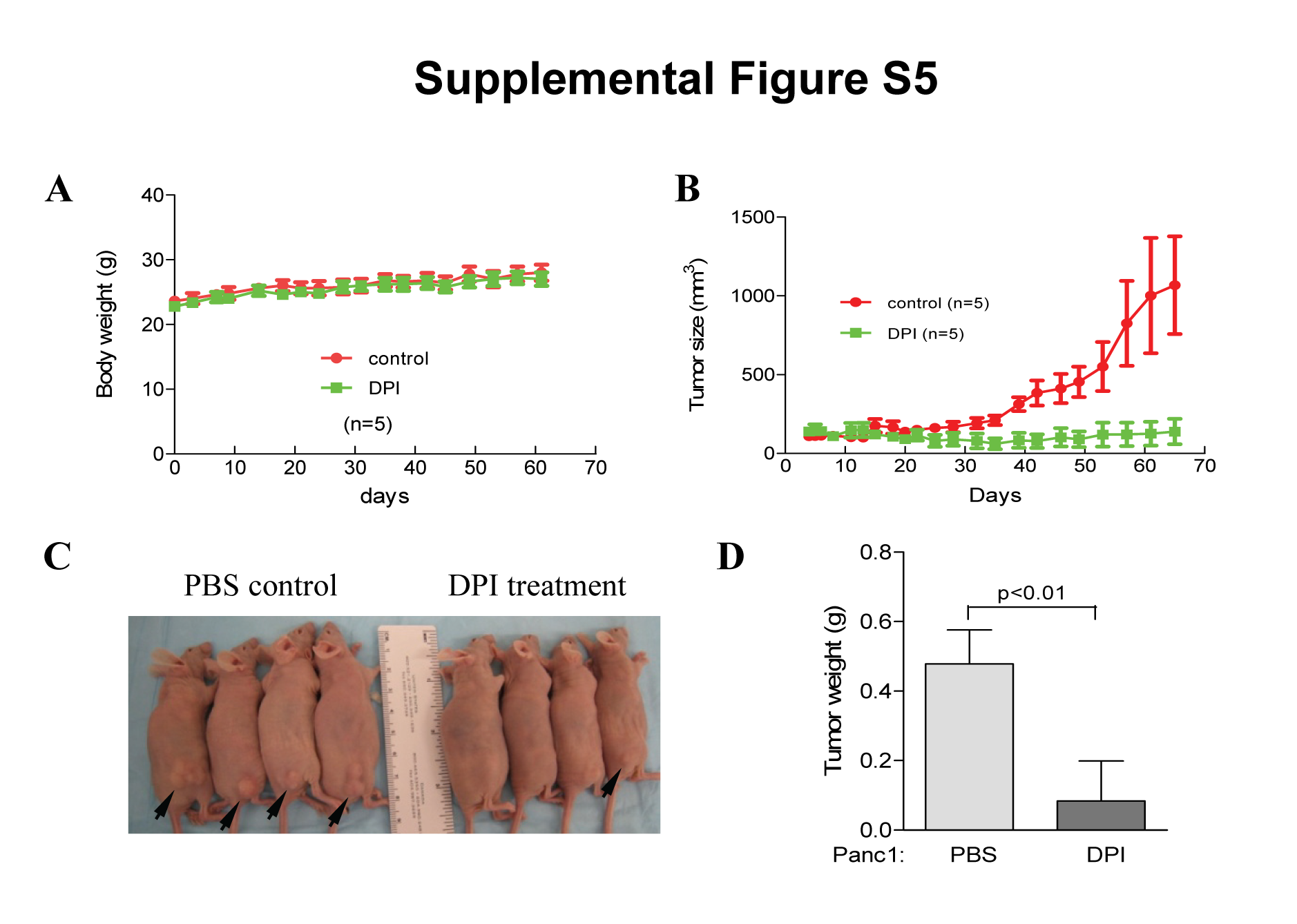

Supplement: Figure S5 — is related to Figure 8. Ten 9-wk-old nude mice were inoculated subcutaneously with Panc-1 cells (5×106/0.15 ml/mouse) and randomly divided into two groups (five mice each). After tumor formation (about 100 mm3), the treatment group received DPI (1.5 mg/kg mouse, i.p.) 5 times per week. The control group received an equal volume of PBS control. The mice were monitored for tumor growth and body weight throughout the experiment. (A) The body weights of the nude mice were measured during the drug treatment to estimate toxicity. Error bars, ±SEM (n = 5). (B) The tumor sizes were measured throughout the experiment to evaluate DPI effect. Data represent tumor volume: mm3±SEM (n = 5). (C) Photographs of nude mice bearing Panc-1 xenografts on day 65 of DPI treatment (one mouse from each group was sacrificed 2 d before). PBS treatment was used as control. (D) Tumor weight derived from control and DPI treated mice on day 65 after DPI treatment was measured. Error bars, ±SEM. p<0.01 and n = 5. (TIF) [file pbio.1001326.s005.tif]
