# Supplementary material for: Novel Role of NOX in Supporting Aerobic Glycolysis in Cancer Cells with Mitochondrial Dysfunction and as a Potential Target for Cancer Therapy
Source: PLoS Biol. 2012 May 8;10(5):e1001326. doi: 10.1371/journal.pbio.1001326 (PMC3348157; doi:10.1371/journal.pbio.1001326)
Supplement: Text S1 — Supplemental materials and methods and supplemental references. (DOC) [file pbio.1001326.s006.doc]

**Text S1: Supplemental Materials and Methods**

**Cell Lines and Cell Culture**

Tet/off T-REx 293 cells bearing POLGdn construction were cultured in the DMEM culture medium supplemented with 10% tetracycline-free FBS in 5% CO_2_ at 37°C in the presence of 100 µg/ml zeocin and 5 µg/ml blasticidin as the selection agents. Tet/on T-Rex 293 cells bearing POLGdn construction were maintained in DMEM supplemented with 10% FBS, 1 mmol/L sodium pyruvate, 50 mmol/L uridine, 0.47% glucose and 100 µg/ml zeocin, 5 µg/ml blasticidin, and 1 µg/ml doxycycline. T72 and T72Ras cells were maintained in MCDB105 and M199 medium mix (1:1) with 15% FBS and 10 ng/ml epidermal growth factor. Before assays, cells were replenished with fresh medium without EGF to eliminate the grow factor effect on the cells. Panc-1 cells were maintained in the DMEM culture medium supplemented with 10% FBS. T-REx 293 cells with long-term induction of K-rasG12V were maintained with 20 ng/ml doxycycline (over 2 months). HPDE cells and HPDE/K-rasG12V cells were cultured in Keratinocyte-SFM medium (GIBCO). HCT116 p53^+/+^ and HCT116 p53^-/-^ colon cancer cells were maintained in the McCoy’s 5A culture medium supplemented with 10% FBS. H1299 and H1299-p53^wt^ cells were maintained in DMEM/F12 50/50 medium supplemented with 10% FBS. To generate H1299-p53^wt^ cell line, H1299 cells were transfected with p53^wt^-pcDNA3.1/Zeocin plasmid as described [1] and stable colonies were selected by Zeocin.

**Reagents**

NADPH, NAD^+^, NADH, lucigenin, propidium iodide (PI), oxypurinol, rotenone, and diphenyleneiodonium (DPI) were purchased from Sigma. ^3^H-2-Deoxyglucose was from Moravek Biochemicals. hydroethidine (HET), MitoSox Red, 2,7-dichlorofluorescin diacetate (DCF-DA), and Rhodamine 123, were from Invitrogen. L-NAME was purchased from Caymen and FITC Annexin V was from BD Biosciences.

**DNA Isolation and Southern Blot Analysis**

DNA was isolated using standard phenol:chloroform:isoamyl alcohol method. For Southern blot assay, 10 µg of genomic DNA including mtDNA from each sample was digested with SphI, separated on 0.8% agarose gel and transferred onto nylon membrane. The probe was generated by random primer labeling (Amersham) of the PCR product of COII gene and used to hybridize the membrane. The mtDNA content can be visualized after washing and exposure the membrane to the X-ray film (Amersham).

**RNA Isolation, Semi-quantitative RT-PCR, and Quantitative Real-time RT-PCR (qRT-PCR)**

Total RNA isolation and cDNA synthesis were carried out as described previously [2]. cDNA was amplified using PCR with a thermocycler. Aliquots of the samples were also tested without reverse transcription to verify that there was no contamination with genomic DNA. The semi-quantitative RT-PCR products were analyzed in agarose gel. The mRNA expression of NOX1, NOX2, NOX4, NOX5, p22phox, p67phox, p47phox, Rac1, Rac2, NOXA1 were analyzed by semi-quantitative RT-PCR or qRT-PCR using following primers: NOX1 (F1410, GGAGCAGGAATTGGGGTCAC, R1645, TTGCTGTCCCATCCGGTGAG) NOX2 (F1136, GGAGTTTCAAGATGCGTGGAAACTA R1685, GCCAGACTCAGAGTTGGAGATGCT); NOX3 (F1326, GGATCGGAGTCACTCCCTTCGCTG, R1783, ATGAACACCTCTGGGGTCAGCTGA); NOX4, (F2039, CTCAGCGGAATCAATCAGCTGTG, R2300, AGAGGAACACGACAATCAGCCTTAG); NOX5 (F1634, ATCAAGCGGCCCCCTTTTTTTCAC, R1849, CTCATTGTCACACTCCTCGACAGC); P47phox (F260, AACAGGATCATCCCCCACCT, R878, CAGGTACATGGACGGAAAGT); p67phox (F332, ATGCCTTCAGTGCCGTCCAG, R758, TGCTTCCAGACACACTCCATCG); p22phox (F45, GATCGAGTGGGCCATGT, R485, TGCTTGATGGTGCCTCC); NOXA1 (F332, CAAGCCGTGACCAAGGACACCTG, R1426, CACACAGGACATCCACCGTGTC); Rac1 (F243, TGCAGGCCATCAAGTGTGTGGT, R830, GCTGAGACATTTACAACAGCAGGCAT); Rac2 (F119, TGCAGGCCATCAAGTGTGTGGT, R695, TAGAGGAGGCTGCAGGCGCGCTT). β-actin (F221 5’GCATCGTCACCAACTGGGAC3’, R740 5’ACCTGGCCGTCAGGCAGCTC3’) was used as internal control. qRT-PCR was performed using SYBR GreenER qPCR supermix (Invitrogen) and an ABI 7900HT machine. For each sample, the expression levels of target gene were normalized using that of β-actin.

**Northern Blot Analysis**

For each sample, 15 µg total RNA (28.38 µg total RNA was loaded at Tet/on d8 sample) was separated by electrophoresis on denaturing formaldehyde gels and transferred onto a nylon membrane. The blot was hybridized with ^32^P-labeled cytochrome c oxidase subunit II (COII) cDNA probe synthesized by RT-PCR. After washing, the membrane was exposed to an X-ray film. The relative level of RNA in each lane was determined by comparing to the 18s and 28s rRNA.

**Western Blot Analysis**

Proteins from whole cell lysates were separated by SDS-PAGE and transferred onto nitrocellulose membranes. The membrane were probed with the following primary antibodies: anti-FLAG (sigma), anti-POLG (Santa Cruz), anti-TFAM (Aviva systems biology), anti-COII (molecular probe), anti-p22phox (Santa Cruz), anti-HKII (Santa Cruz), anti-SOD1 (Calbiochem), anti-SOD2 (Lab Frontier), and anti-β-actin (Sigma). The bound primary antibodies were then probed with their respective secondary antibodies conjugated to horseradish peroxidase. Immunolabeled proteins were detected by using a SuperSignal enhanced chemiluminescence kit (Pierce, Rockford, IL) and autoradiography. For sequential blotting, the membranes were stripped by using stripping buffer (Pierce) and reprobed with other antibodies or β-actin specific antibody to provide a loading control.

**siRNA and shRNA Transfection**

Cells were transiently transfected with double-stranded RNA oligonucleotides against p22phox, NOX1 or scrambled scRNA (Dharmacon SMART pool) using lipofectamine 2000 reagent according to the manufacturer's instructions (Invitrogen). Functional effects on the target gene knockdown were analyzed after 72-96 hr transfection. For stable knockdown, p22phox shRNA lentiviral particles and control shRNA lentiviral particles were purchased from Santa Cruz. Infection and colony selection by puromycin are based on manufacturer’s protocol.

**Subcellular Fractionation of Membrane-associated Fraction**

Cells were washed with cold PBS and collected by scratching in cold PBS and centrifugation at 1400 rpm for 5 min. Cells were then resuspended in cold HEPES buffer (20 mM HEPES, 10 mM KCl, 1.5 mM MgCl_2_, 1 mM EGTA, 1 mM EDTA, and protease inhibitor cocktail) and homogenized using a tight-fight glass tissue homogenizer for 12-14 strokes. Cell nuclei and unbroken cells were removed by centrifugation of the homogenates at 1300x g at 4°C for 5 min twice. The resulting supernatant was further centrifuged at 100,000x g for 40 min at 4°C to separate cytosolic fraction and the membrane-associated fraction (including mitochondrial membrane). The membrane-associated fraction was resuspended in buffer containing 50 mM Tris-HCl, 150 mM NaCl, 1 mM EDTA. Protein concentration was determined using a BCA protein assay kit from Pierce.

**Assay of *in vivo* Antitumor Activity of NOX Inhibitor DPI**

Ten nude mice were injected subcutaneously with Panc-1 cells (5 × 10^6^ cells/mouse) and randomly divided into two groups (5 mice each). When the tumor volumes reached 100 mm^3^, intraperitoneal injections with DPI (1.5mg/kg mouse) or vehicle control PBS were initiated and carried out 5 days per week for 65 days. Tumor size and body weight were measured throughout the experiment.

**Colony Formation Assay**

Soft agar colony formation assays were done in six-well dishes. Briefly, Panc-1 cells (1 × 10^4^) transfected with either control shRNA plasmid or p22phox-shRNA plasmid were suspended in 0.35% agar with growth medium (DMEM supplemented with 10% FBS). Cells were added to a base layer of 0.7% agar containing the same culture medium and incubated at 37°C in a 5% CO_2_ incubator for 2 weeks. Colony formation was assessed by counting the number of colonies on each well.

**NADPH and NADP Measurement**

Cellular NADPH and NADP of Tet/off and Tet/on (day 8) cells were measured using fluoroscent NADP/NADPH detection kit from Cell Technology according to the manufacturer’s instructions. NADPH/NADP ratio was calculated.

**Supplemental References**

1. Achanta G, Sasaki R, Feng L, Carew JS, Lu W, et al. (2005) Novel role of p53 in maintaining mitochondrial genetic stability through interaction with DNA Pol gamma. Embo J 24: 3482-3492.

2. Lu W, Luo Y, Kan M, McKeehan WL (1999) Fibroblast growth factor-10. A second candidate stromal to epithelial cell andromedin in prostate. J Biol Chem 274: 12827-12834.
