# Supplementary material for: Correction: Novel Role of NOX in Supporting Aerobic Glycolysis in Cancer Cells with Mitochondrial Dysfunction and as a Potential Target for Cancer Therapy
Source: PLoS Biol. 2017 Dec 11;15(12):e1002616. doi: 10.1371/journal.pbio.1002616 (PMC5724824; doi:10.1371/journal.pbio.1002616)
Supplement: S1 File — POLGdn cells were induced by doxycycline for various times (Tet/on, 1–16 days) as indicated. The cell lysates were then analyzed for expression of HKII protein by western blotting. β-actin was also blotted as a protein loading control. For quantitative analysis, the band density for each time point was first divided by the band density of the Tet/off control band (0 day) and expressed as a relative value (fold). The value represented by each HKII band was then further normalized by the corresponding β-actin band value. The detailed quantitation data were shown in Supplemental Table 1 on the next page. (PDF) [file pbio.1002616.s001.pdf]

Replicate #1:

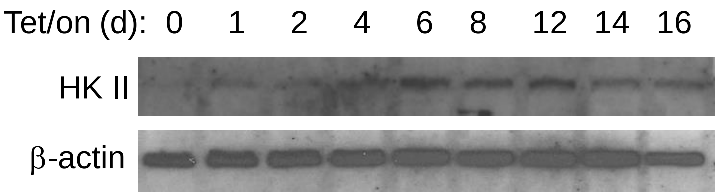

Replicate #2:

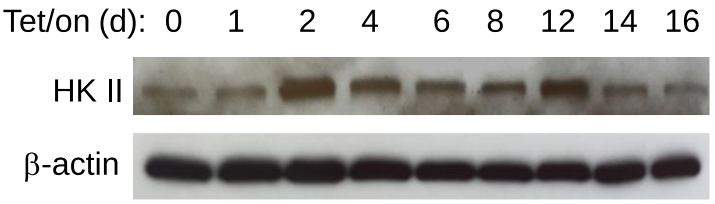

Replicate #3:

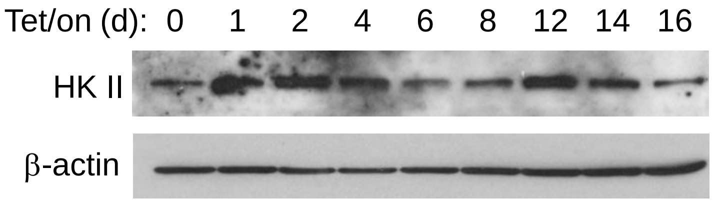

Supplemental Table 1: Quantitative analysis of band density of HKII normalized by the corresponding β-actin for replicate gels #1-3

	**Time (day)**	**0**	**1**	**2**	**4**	**6**	**8**	**12**	**14**	**16**
**Replicate #1**	HKII density	4715.761	7644.945	5871.673	7400.472	15235.309	14468.622	13706.087	13038.966	12646.614
	HKII density relative to day 0	1.0	1.621147679	1.245116748	1.569305993	3.230721192	3.068141494	2.906442248	2.764976003	2.681775858
	β-Actin density	22256.182	25843.726	25925.082	26727.839	25651.052	26234.798	26271.408	27453.91	21968.999
	β-Actin density relative to day 0	1.0	1.161193251	1.164848685	1.200917633	1.15253615	1.17876464	1.180409576	1.233540976	0.987096573
	**Normalized HKII (fold change)**	1.0	1.396104979	1.068908575	1.306755726	2.803140876	2.602844869	2.462232014	2.241495059	2.716832306
**Replicate #2**	HKII density	13624.572	12422.865	41831.241	28305.17	21329.622	17628.572	23342.099	13707.848	14244.865
	HKII density relative to day 0	1.0	0.91179855	3.070279272	2.07750893	1.565526022	1.293880791	1.713235396	1.006112192	1.045527522
	β-Actin density	21131.853	17100.543	13237.803	14059.966	10722.267	8482.589	9501.61	13937.48	14751.004
	β-Actin density relative to day 0	1.0	0.809230758	0.626438433	0.665344776	0.507398406	0.401412512	0.449634556	0.659548501	0.69804603
	**Normalized HKII (fold change)**	1.0	1.126747273	4.901166835	3.122454712	3.085397991	3.223319531	3.810284088	1.525455959	1.49779166
**Replicate #3**	HKII density	11032.693	13637.304	38129.65	16729.057	20182.827	22660.362	41461.588	30364.011	28661.709
	HKII density relative to day 0	1.0	1.236081164	3.456060093	1.516316732	1.829365414	2.053928447	3.758065959	2.752184893	2.597888748
	β-Actin density	14714.359	15953.945	11715.681	10265.903	12582.217	15523.187	17648.137	19122.451	15854.622
	β-Actin density relative to day 0	1.0	1.084243215	0.79620731	0.697679206	0.855097809	1.054968548	1.199381896	1.299577488	1.077493143
	**Normalized HKII (fold change)**	1.0	1.140040487	4.340653556	2.173372403	2.139363935	1.946909651	3.133335571	2.117753592	2.411048984

Note: See the figure legend for Supplemental Data on the previous page for detail experimental procedures.
